# Gut microbiota intervention attenuates thermogenesis in broilers exposed to high temperature through modulation of the hypothalamic 5-HT pathway

**DOI:** 10.1186/s40104-023-00950-0

**Published:** 2023-12-21

**Authors:** Sheng Li, Xiaoqing Li, Kai Wang, Yansen Li, Kentaro Nagaoka, Chunmei Li

**Affiliations:** 1 Research Centre for Livestock Environmental Control and Smart Production, College of Animal Science and Technology, Nanjing Agricultural University, Nanjing, 210095 China; 2 Laboratory of Veterinary Physiology, Department of Veterinary Medicine, Faculty of Agriculture, Tokyo University of Agriculture and Technology, Tokyo, 183-8509 Japan

**Keywords:** 5-HT, Broiler chickens, Gut microbiota, Thermogenesis, Thermoregulation

## Abstract

**Background:**

Broilers have a robust metabolism and high body temperature, which make them less tolerant to high-temperature (HT) environments and more susceptible to challenges from elevated temperatures. Gut microbes, functioning as symbionts within the host, possess the capacity to significantly regulate the physiological functions and environmental adaptability of the host. This study aims to investigate the effects of gut microbial intervention on the body temperature and thermogenesis of broilers at different ambient temperatures, as well as the underlying mechanism involving the "gut-brain" axis.

**Methods:**

Broilers were subjected to gut microbiota interference with or without antibiotics (control or ABX) starting at 1 day of age. At 21 day of age, they were divided into 4 groups and exposed to different environments for 7 d: The control and ABX groups at room temperature (RT, 24 ± 1 °C, 60% relative humidity (RH), 24 h/d) and the control-HT and ABX-HT groups at high temperature (HT, 32 ± 1 °C, 60% RH, 24 h/d).

**Results:**

The results demonstrated that the antibiotic-induced gut microbiota intervention increased body weight and improved feed conversion in broiler chickens (*P* < 0.05). Under HT conditions, the microbiota intervention reduced the rectal temperature of broiler chickens (*P* < 0.05), inhibited the expression of *avUCP* and thermogenesis-related genes in breast muscle and liver (*P* < 0.05), and thus decreased thermogenesis capacity. Furthermore, the gut microbiota intervention blunted the hypothalamic‒pituitary‒adrenal axis and hypothalamic–pituitary–thyroid axis activation induced by HT conditions. By analyzing the cecal microbiota composition of control and ABX chickens maintained under HT conditions, we found that *Alistipes* was enriched in control chickens. In contrast, antibiotic-induced gut microbiota intervention resulted in a decrease in the relative abundance of *Alistipes* (*P* < 0.05). Moreover, this difference was accompanied by increased hypothalamic 5-hydroxytryptamine (5-HT) content and *TPH2* expression (*P* < 0.05).

**Conclusions:**

These findings underscore the critical role of the gut microbiota in regulating broiler thermogenesis via the gut-brain axis and suggest that the hypothalamic 5-HT pathway may be a potential mechanism by which the gut microbiota affects thermoregulation in broilers.

**Graphical Abstract:**

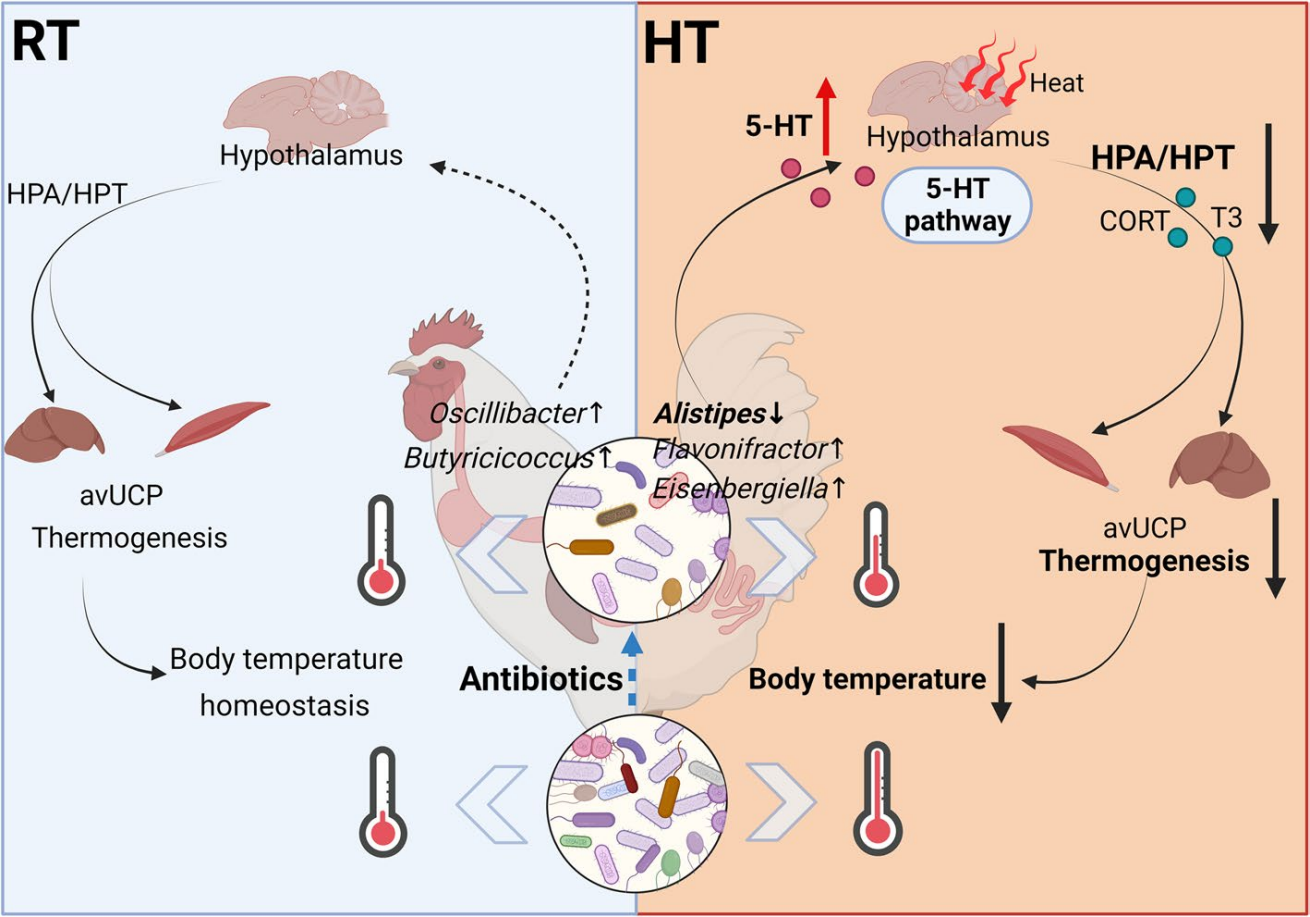

**Supplementary Information:**

The online version contains supplementary material available at 10.1186/s40104-023-00950-0.

## Introduction

Accumulating evidence suggests that the gut microbiota plays a crucial role in regulating the metabolic homeostasis of the host. Along with the host's own physiological and behavioral strategies, the complex commensal microbiota inhabiting the gut can significantly influence the host's phenotype and fitness in response to environmental temperature changes. Previous studies have demonstrated that exposure to cold triggers significant alterations in the composition of the mouse microbiota [1]. Transplantation of this "cold microbiota" into germ-free mice is sufficient to induce white fat browning and partial cold tolerance in the recipients [1, 2]. In contrast, in germ-free or antibiotic-treated mice, the browning of white fat and expression of uncoupling protein 1 (UCP1) in brown fat were inhibited, leading to impaired thermogenesis [3, 4]. Further investigation of the role of the intestinal microbiota as a cold-induced thermogenesis system revealed that a transplanted cold-adapted gut microbiota interacts with neurotransmitters to promote thermogenesis in Brandt's voles [2]. Furthermore, several studies have shown that unique microbiomes shaped by changes in the gut environment result in distinct thermogenic and metabolic phenotypes [5, 6]. Research involving Tibetan chickens has elucidated that their unique gut microbiota composition endows them with the capacity to effectively regulate their body temperature in environments characterized by low oxygen levels and extreme cold [7]. Significantly, these chickens also demonstrate the ability to adapt to new environments by dynamically reshaping their gut microbiota, which further underscores the critical role of the gut microbiota in facilitating thermoregulatory adaptability [7].

Body temperature homeostasis is vital and requires precise regulation by the nervous system. Numerous studies have substantiated the presence of a bidirectional signaling pathway between gut microbes and the host brain, commonly termed the "gut-brain" axis. Through this axis, gut microbes can influence various physiological functions of the host brain, including neuron development, host behavior, learning, memory, appetite, and emotional regulation [8–10]. Increasing evidence suggests that the gut microbiota is closely linked to hypothalamic thermoregulation. Recent findings have shown that muropeptide, a component of intestinal bacterial cell walls, can accumulate in the brain via the blood circulation and directly inhibit hypothalamic neuronal activity, leading to decreased body temperature in mice [11, 12]. Furthermore, various neurotransmitters produced by gut microbes, such as 5-HT and GABA [13, 14], as well as metabolites, such as butyrate and succinate [4, 15], have been reported to influence hypothalamic neurons and contribute to thermoregulation.

To date, our understanding of the relationship between the gut microbiota and thermoregulation is primarily based on research in mammals, and little is known about the mechanisms involved in other endothermic animals, such as chickens. Broiler chickens, which have a high metabolic rate, high body temperature, and poor cooling capacity, provide a unique model to investigate the regulation of thermogenesis. Notably, broilers face greater thermoregulatory demand due to the presence of feathers and a lack of sweat glands and are more susceptible to heat stress [16]. The hypothalamus serves as the principal thermostat of broilers and is capable of receiving environmental temperature information, thus exerting a profound influence on broiler body temperature regulation [17]. More so, during the maturation of the broiler thermoregulatory system, the ability to maintain homeostatic body temperature frequently hinges on alterations in the heat sensitivity of the hypothalamus [18].

Based on the central role of the hypothalamus in thermogenesis and thermoregulation in chickens, we speculated that gut microbes may regulate thermogenesis and body temperature in broiler chickens via the gut-brain axis. To confirm this hypothesis, in this study, we induced depletion and disruption of the broilers' gut microbiota by adding broad-spectrum antibiotics to the drinking water. Subsequently, we investigated the impact of cecal microbiota intervention on skeletal muscle and liver thermogenesis, as well as the thermogenesis regulation pathways and mechanisms in the hypothalamus.

## Materials and methods

All research procedures were approved by the Nanjing Agricultural University Animal Care and Use Committee (permit number SYXK (Su) 2021–0036) and complied with the Regulations on the Administration of Laboratory Animals promulgated by the National Science and Technology Commission of the People's Republic of China (Beijing).

### Birds and care

Two hundred fertilized eggs (Arbor Acres, *Gallus gallus domesticus*) were purchased from a hatchery in Jiangsu, China. The incubation temperature was 38 °C, with 60%–70% humidity and autoturning every 1.5 h in the incubator. After hatching, all chicks were transferred and reared in environmentally controlled chambers. The ambient temperature was 35 °C for the first 2 d and then gradually reduced to 24 °C until 21 d. Artificial lighting was continuous before 2 d, and afterward, a 23 L:1 D schedule was applied during the entire rearing period. Chicks were fed a commercial starter diet (21.0% CP and 2,900 kcal/kg ME from 1 to 21 d) or grower diet (19.0% CP and 3,030 kcal/kg of ME from 22 to 28 d) until the end of the experiment. The diet formula is shown in Table S1 (Additional file 1). Feed was offered ad libitum in pelleted form, and water was available at all times.

### Gut microbiota intervention

At 1 day of age, 96 healthy broilers with similar body weight (approximately 52 g) were selected and randomly assigned to 2 groups, and each group had 4 pens of 12 birds. The chicks were provided drinking water with (ABX) or without (control) antibiotics from 1 to 28 days of age. The ABX protocol was prepared based on previous research protocols in mice [3], with suitable adjustments, so the administered solution contained neomycin (1 mg/mL, J&K, Beijing, China), gentamicin (1 mg/mL, J&K, Beijing, China), streptomycin (1 mg/mL, Sigma, Saint Louis, MO, USA), and amikacin (1 mg/mL, Sigma, Saint Louis, MO, USA). From 1 to 21 days of age, the body weight and rectal temperature of the broilers were recorded weekly. At 21 days of age, cecal content from the broilers was collected to assess the effectiveness of gut microbiota intervention induced by the ABX protocol.

### Experimental design

Broilers in the control and ABX groups were reared in environmentally controlled chambers at the same ambient temperature until 21 days of age. At 21 days of age, 64 healthy broilers from both groups were selected and assigned to 4 groups, and each group had 4 pens of 4 birds. Broilers in the control-HT and ABX-HT groups were shifted and housed in high-temperature (HT) chambers at 32 ± 1 °C and in 60% RH 24 h/d from d 22 to 28. Broilers in the control-RT and ABX-RT groups were reared in room-temperature (RT) chambers at 24 ± 1 °C and in 60% RH 24 h/d from d 22 to 28. During the entire experimental period, rectal temperatures were taken individually under a fasting state simultaneously every week.

### Sample collection

At 21 and 28 days of age, feed intake and body weight gain were recorded per cage to calculate feed conversion efficiency (FCR, ADFI/ADG). After a 12 h overnight fast [19], 8 broilers in each treatment group were randomly selected and euthanized. Euthanasia was conducted through CO_2_ asphyxiation, followed by exsanguination. The breast muscle (both the pectoralis major and minor) and liver were sampled and weighed individually. Moreover, the hypothalamus and cecal contents were harvested and immediately frozen in liquid nitrogen for future analysis.

### Body temperature measurement

The rectal temperature was measured using a Thermalert monitoring thermometer (TH-5, Physitemp, Clifton, NJ, USA). The thermometer probe was inserted into the rectum at a 2–3 cm depth, and then the data from the thermometer were recorded 2 s later. The overall accuracy of the measuring system was ± 0.1 °C.

### Serum parameters

Blood samples were collected from the wing vein and allowed to naturally coagulate. Subsequently, they were centrifuged at 3,000 × *g* for 10 min to collect the serum, which was then stored at −20 °C for further analysis. Serum triiodothyronine (T3) and thyroxine (T4) concentrations were quantified by radioimmunoassay using Iodine (^125^I) thyroxine radioimmunoassay kit and Iodine (^125^I) 3,3’,5-triiodothyronine radioimmunoassay kit (Beijing North Institute of Biotechnology Co., Ltd., Beijing, China) according to the manufacturer's instructions. Serum corticosterone and 5-HT concentrations were determined spectrophotometrically (Spark Absorbance Microplate Reader, Tecan, Männedorf, Switzerland) with chicken corticosterone ELISA kit and chicken 5-HT ELISA kit (Shanghai Enzyme-linked Biotechnology Co., Ltd., Shanghai, China).

### Hypothalamic neurotransmitters

Hypothalamic 5-HT concentrations were measured spectrophotometrically with commercial ELISA kits (Nanjing Jiancheng Bioengineering Institute Co., Ltd., Nanjing, China) according to methods from previous studies [20]. The total protein concentration of the homogenate was measured by bicinchoninic acid (BCA) assay using bovine serum albumin as the standard.

### RT-qPCR

Total RNA was extracted from breast muscle and liver tissue using TRIzol reagent (Invitrogen, Carlsbad, CA, USA). The concentration of the RNA was measured by spectrophotometry (Thermo Fisher Scientific, Waltham, MA, USA). Next, reverse transcription was performed using total RNA (1 μg) for first-strand cDNA synthesis with the Transcriptor First Strand cDNA Synthesis Kit (ABclonal, Wuhan, China). The cDNA was amplified in a 20 μL PCR system containing 0.2 μmol/L each specific primer (Sangon, Shanghai, China) and SYBR Green master mix (ABclonal, Wuhan, China) according to the manufacturer's instructions. Real-time PCR was performed on an ABI QuantStudio 7 PCR machine (Applied Biosystems; Thermo Fisher Scientific, Waltham, MA, USA), and the primer sequences are shown in Table S2 (Additional file 2).

The absolute abundance of total bacteria was analyzed according to methods from a previous study [21]. The total DNA of the microbiota from cecal contents was extracted using the cetyltrimethylammonium bromide (CTAB) method. The concentration of the DNA was measured by spectrophotometry. The DNA from the standards and samples was amplified in a 20 μL PCR system with total bacterial-specific primers (F: 5'-GTGSTGCAYGGYYGTCGTCA-3'; R: 5'-ACGTCRTCCMCNCCTTCCTC-3') and SYBR Green master mix according to the manufacturer's instructions. A standard curve was generated from pure cultures of *Escherichia coli* and used to calculate total bacterial contents.

### 16S rRNA gene amplicon sequencing

Total genomic DNA from cecal contents was extracted and stored at −20 °C for 16S rRNA gene amplicon sequencing. The V3–V4 hypervariable region of 16S rRNA genes was amplified using specific primers (338F, 5'-ACTCCTACGGGAGGCAGCAG-3'; 806R, 5'-GGACTACHVGGGTWTCTAAT-3') with a barcode. The same volume of 1× loading buffer (containing SYBR Green) with PCR products was mixed and operated with 2% agarose gel for detection. The products were quantified using a Qubit 3.0 Fluorometer (Thermo Fisher Scientific, Waltham, MA, USA), screened and used to construct sequence libraries. Finally, the library was sequenced on an Illumina HiSeq 2500 platform, and 250-bp paired-end reads were generated. Sequences with 97% similarity were clustered into the same operational taxonomic units (OTUs). The alpha and beta diversity were calculated with QIIME (version 1.9.1) and displayed by using R software (version 2.15.3). Alpha diversity indices, including the Sobs, Chao1, ACE, Shannon, Simpson and Pielou indices, were applied to analyze the species diversity complexity for each sample. Beta diversity was analyzed through principal coordinates analysis (PCoA) and nonmetric multidimensional scaling (NMDS) to evaluate differences between samples in species complexity. Total genomic DNA extraction, PCR amplification, sequencing, and data quality control were performed by Gene Denovo Co., Ltd. (Guangzhou, China).

### Statistical analysis

Data are expressed as the mean ± SEM. Data collected were analyzed with Student's *t-*test or two-way ANOVA using Statistical Analysis System software (version 8e; SAS Institute, Cary, NC, USA). Two-way ANOVA was employed to analytically assess the main effects (temperature and ABX) and their interactions. When the main effects or their interaction was found to be significant, one-way ANOVA was applied. Mean separation was performed using Duncan’s multiple comparisons, and treatment effects were considered statistically significant at a probability of *P* < 0.05.

For the relative abundance of the cecal microbiota at the phylum and genus levels, the Tukey HSD-test and Welch's *t-*test were used for statistical analysis, and *P* < 0.05 was considered to indicate a significant difference. Raw data analysis of 16S rRNA gene amplicon sequencing was performed on the Omicsmart platform (Gene Denovo Co., Ltd., Guangzhou, China).

## Results

### Effect of the ABX protocol on the performance and total bacterial content in the cecum of broilers

**Fig. 1:**
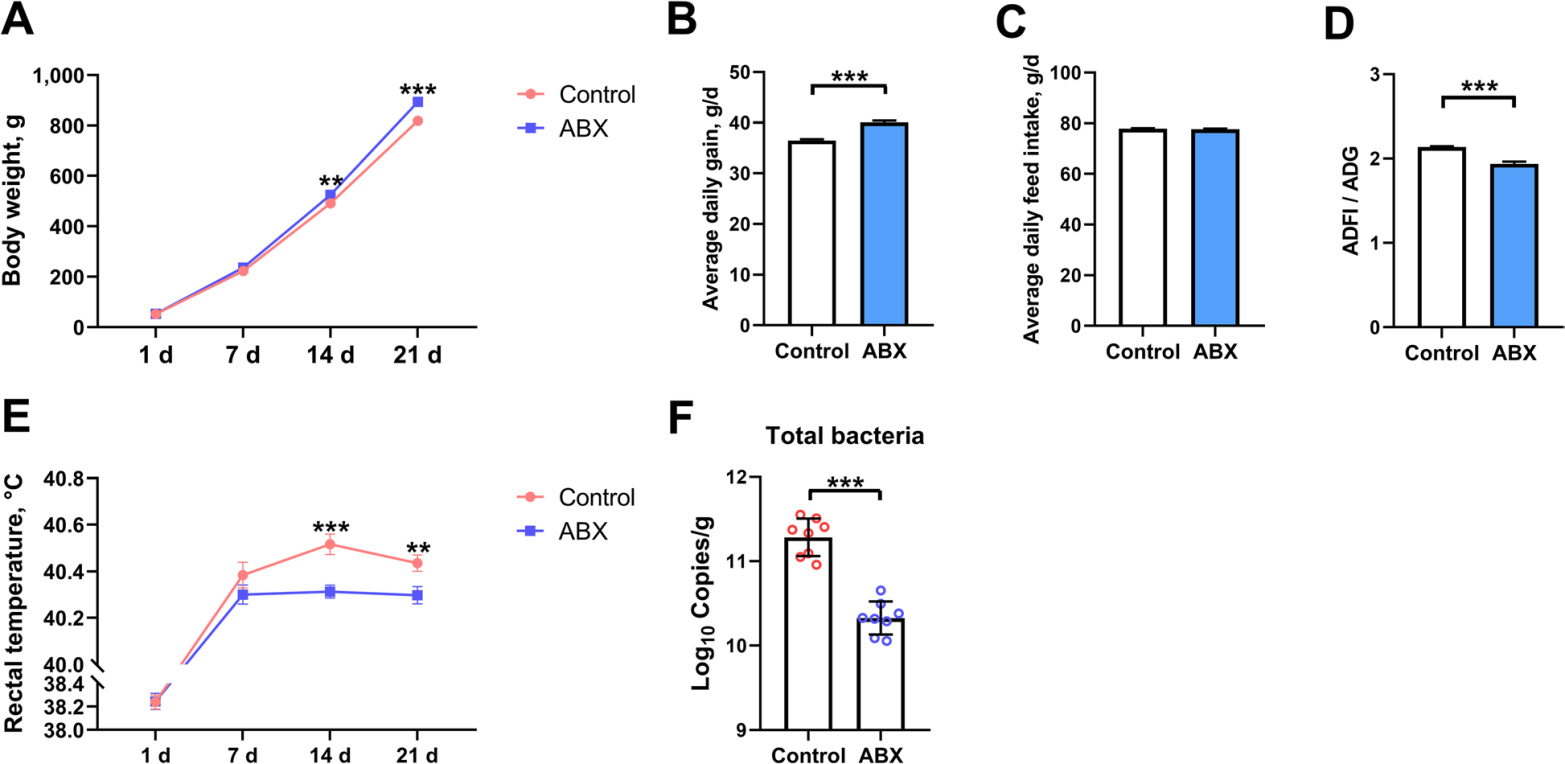
Effect of the ABX protocol on the performance and total bacterial content in the cecum of broilers from 1 to 21 d. **A** The changes in body weight of broilers from 1 to 21 d. **B** Average daily gain. **C** Average daily feed intake. **D** Feed conversion ratio. **E** The changes in the rectal temperature of broilers from 1 to 21 d (*n =* 32). **F** The abundance of total bacteria in cecal contents of broilers at 21 days of age (*n =* 8). Data are expressed as the mean ± SEM. ^*^P < 0.05, ^**^P < 0.01, ^***^P < 0.001

The body weight and rectal temperature of the broilers from 1 to 21 days of age were recorded weekly (Fig. 1). After 14 d of ABX treatment, the mean body weight was significantly higher (*P* = 0.006) in the ABX group, while the rectal temperature was lower at d 14 (*P* < 0.001) and 21 (*P* = 0.008) compared to those in the control group. At d 21, ABX-treated broilers showed higher (*P* < 0.001) body weight and average daily gain (ADG) and a lower ADFI/ADG ratio, with no significant difference (*P* = 0.402) in average daily feed intake (ADFI) compared to those of the control broilers. Total bacterial contents were measured and used to evaluate the efficiency of ABX-mediated depletion of the gut microbiota. Quantitative results showed that ABX treatment significantly reduced (*P* < 0.001) the bacterial content in the cecum at 21 days of age.

### Effect of gut microbiota intervention on the performance and thermoregulation of broilers under different ambient temperatures

**Fig. 2:**
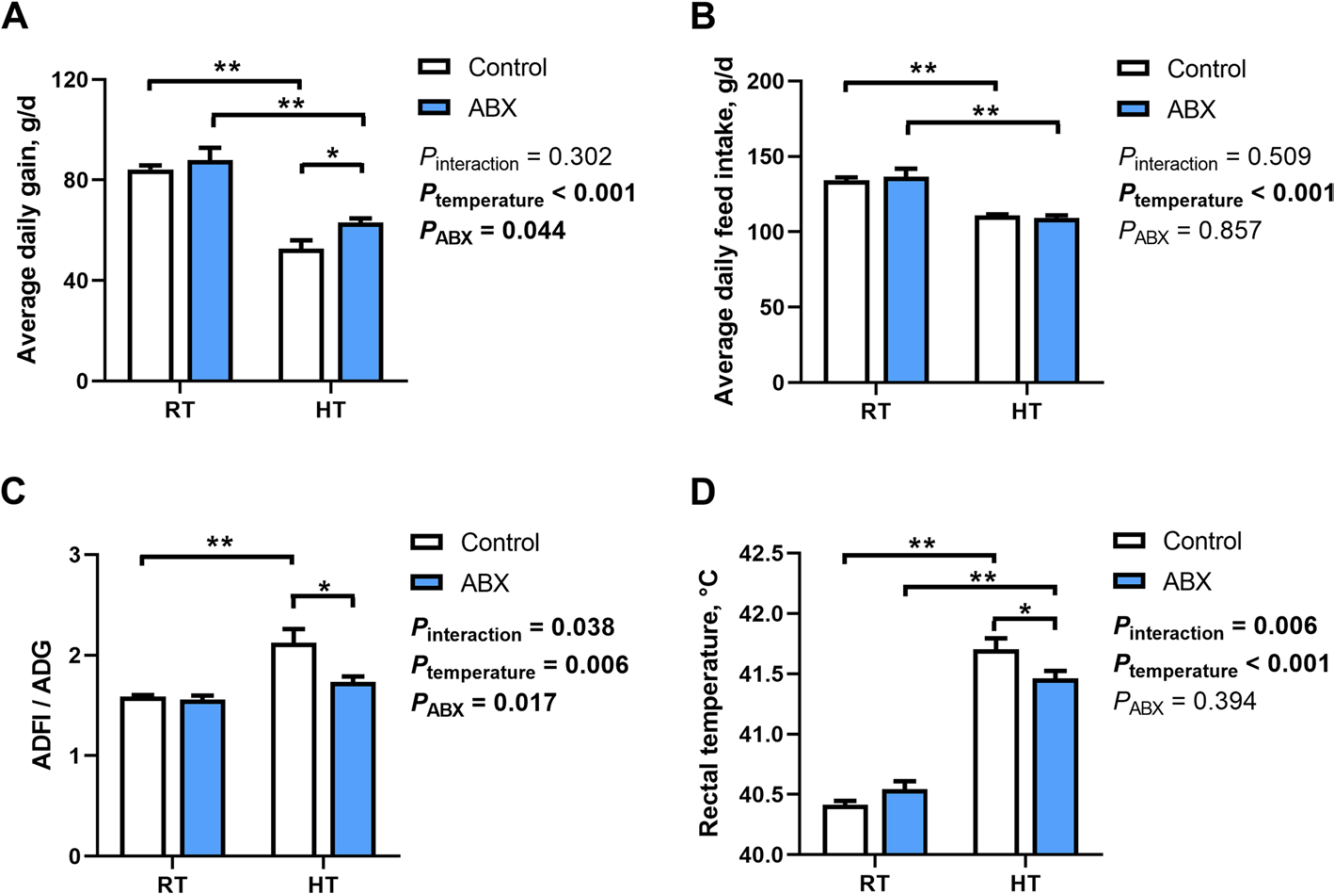
Effect of gut microbiota intervention on performance and body temperature of broilers under different temperature conditions. **A** Average daily gain. **B** Average daily feed intake. **C** Feed conversion ratio. **D** The rectal temperature of broilers (*n =* 16). Data are expressed as the mean ± SEM. ^*^P < 0.05, ^**^P < 0.01, ^***^P < 0.001

Models for different ambient temperatures were constructed to investigate the effect of gut microbiota intervention on broiler thermoregulation (Fig. 2). ADG and ADFI were reduced (*P*_temperature_ < 0.001) in broilers exposed to HT compared to those in broiler maintained at RT. However, the ADFI/ADG ratio was elevated only in the control broilers (*P* = 0.002). Under HT conditions, ABX treatment increased (*P* = 0.028) the ADG and decreased (*P* = 0.017) the ADFI/ADG ratio compared with those under control conditions. The rectal temperature was higher (*P*_temperature_ < 0.001) in broilers exposed to HT than in those exposed to RT. Moreover, ABX-treated chickens exhibited lower (*P* = 0.049) rectal temperature than control chickens under HT conditions but not under RT conditions.

### Effect of gut microbiota intervention on thermogenesis in the liver and breast muscle of broilers under different ambient temperatures

**Fig. 3:**
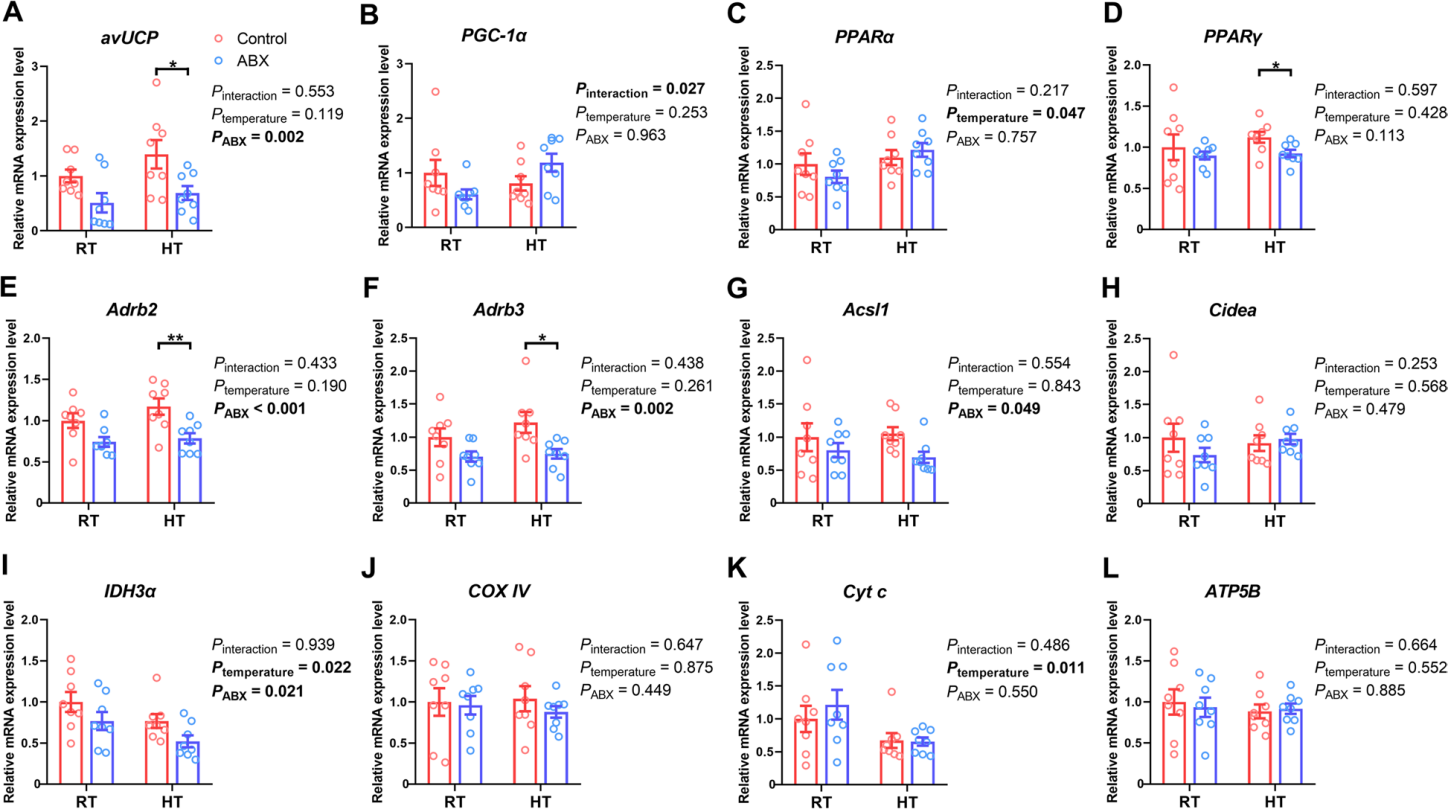
Effect of gut microbiota intervention on the expression of genes associated with thermogenesis (**A–H**) and oxidative phosphorylation (**I–L**) in the liver under different ambient temperatures. Data are expressed as the mean ± SEM (*n =* 8). ^*^P < 0.05, ^**^P < 0.01, ^***^P < 0.001

Exposure to HT appeared to significantly increase the expression of *PPARα* (*P*_temperature_ = 0.047) and decrease the expression of *IDH3α* (*P*_temperature_ = 0.022) and *Cyt c* (*P*_temperature_ = 0.011) in the liver (Fig. 3). Compared with the control, ABX treatment significantly reduced the expression of *avUCP* (*P*_ABX_ = 0.002), *Adrb2* (*P*_ABX_ < 0.001), *Adrb3* (*P*_ABX_ = 0.002), *Acsl1* (*P*_ABX_ = 0.049), and *IDH3α* (*P*_ABX_ = 0.021). Under RT conditions, there was no significant difference in the expression of genes related to thermogenesis and mitochondrial function between the control and ABX groups (*P* > 0.05). Under HT conditions, ABX treatment significantly inhibited the expression of thermogenesis genes, including *avUCP* (*P* = 0.044), *PPARγ* (*P* = 0.026)*, Adrb2* (*P* = 0.009), and *Adrb3* (*P* = 0.034), without affecting (*P* > 0.05) mitochondrial gene expression.

**Fig. 4:**
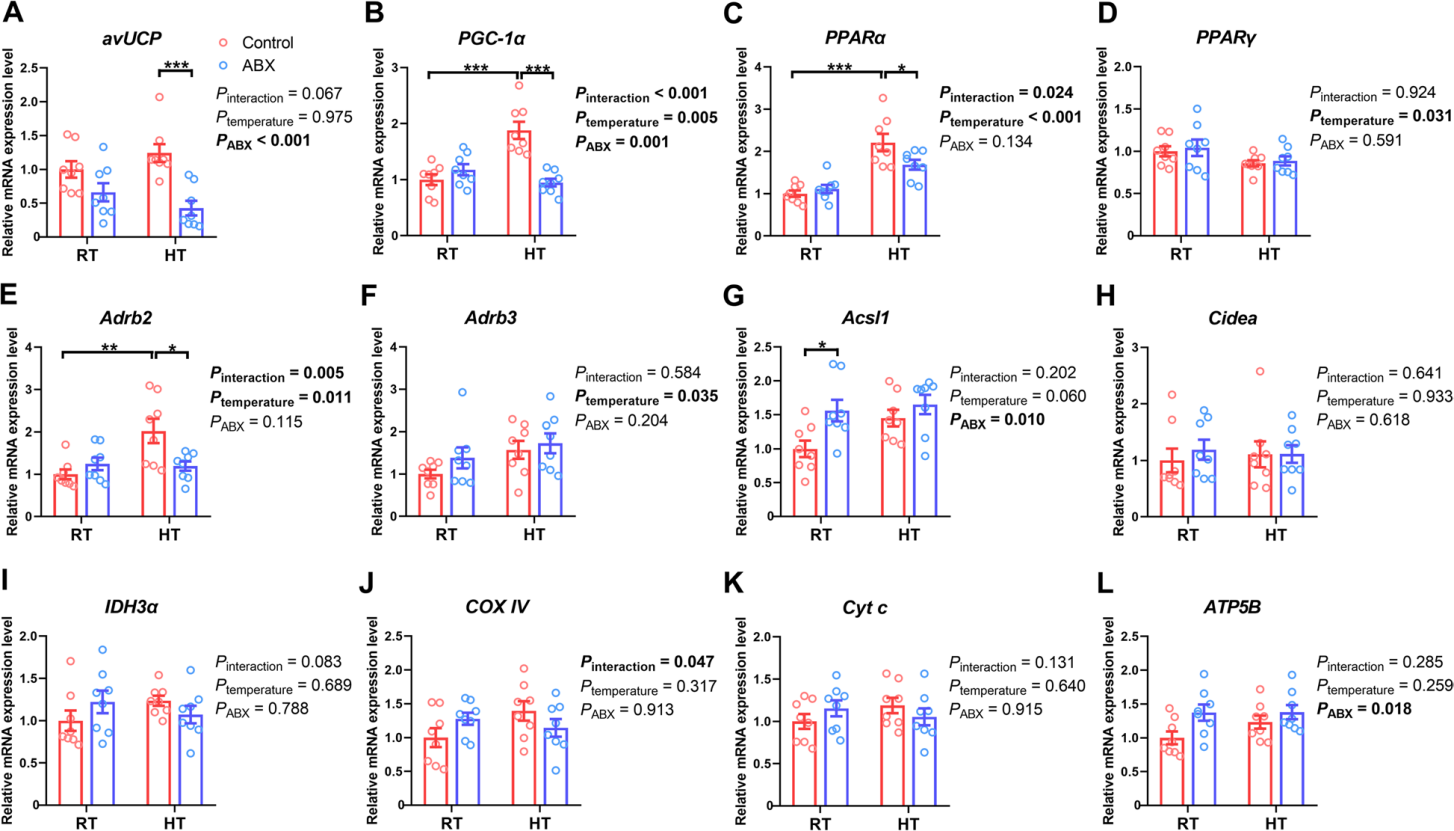
Effect of gut microbiota intervention on the expression of genes associated with thermogenesis (**A–H**) and oxidative phosphorylation (**I–L**) in the breast muscle under different ambient temperatures. Data are expressed as mean ± SEM (*n =* 8). ^*^P < 0.05, ^**^P < 0.01, ^***^P < 0.001

Compared with RT conditions, HT exposure increased the expression of *PGC-1α* (*P*_temperature_ = 0.005), *PPARα* (*P*_temperature_ < 0.001), *Adrb2* (*P*_temperature_ = 0.011) and *Adrb3* (*P*_temperature_ = 0.035) while decreasing the expression of *PPARγ* (*P*_temperature_ = 0.031) in the breast muscle of broilers (Fig. 4). Specifically, HT exposure increased the expression of *PGC-1α* (*P* < 0.001), *PPARα* (*P* < 0.001), and *Adrb2* (*P* = 0.002) in control chickens but did not affect that in ABX chickens. Compared with the control, ABX treatment led to a significant reduction in the expression of *avUCP* (*P*_ABX_ < 0.001) and *PGC-1α* (*P*_ABX_ = 0.001), while resulting in increased expression of *Acsl1* (*P*_ABX_ = 0.010) and *ATP5B* (*P*_ABX_ = 0.018). Under RT conditions, only *Acsl1* mRNA expression was upregulated (*P* = 0.036) in ABX-treated chickens compared with those of control chickens. In contrast, the expression levels of *avUCP* (*P* < 0.001), *PGC-1α* (*P* < 0.001), *PPARα* (*P* = 0.045), and *Adrb2* (*P* = 0.014) were notably lower in ABX-treated chickens under HT conditions. However, under the same conditions, ABX treatment did not influence (*P* > 0.05) the expression level of mitochondrial respiratory chain genes in the breast muscle.

### Effect of gut microbiota intervention on gene expression and hormone levels related to thermoregulation in broilers under different ambient temperatures

**Fig. 5:**
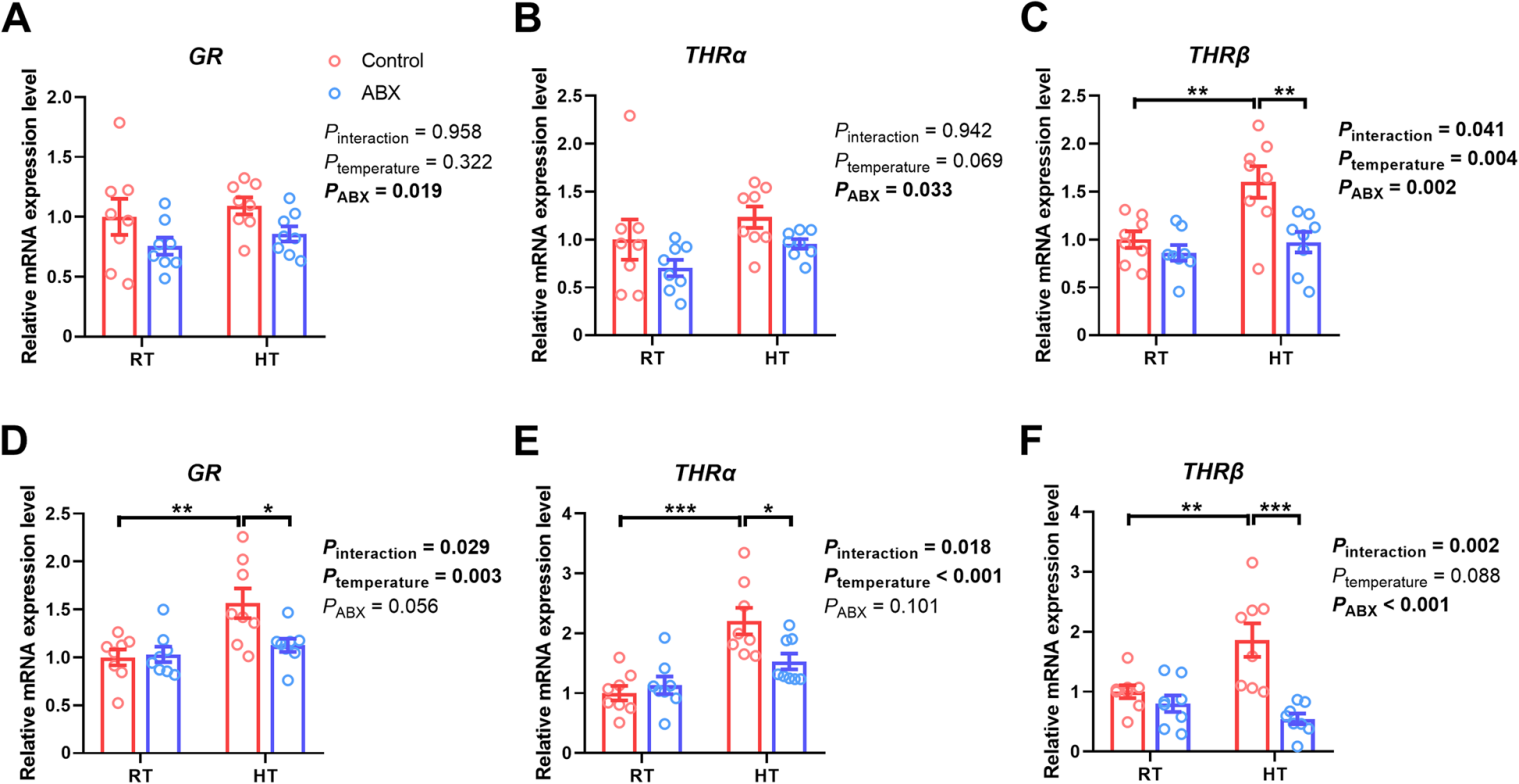
Effect of gut microbiota intervention on the expression of genes associated with hormone receptors in the liver (**A–C**) and breast muscle (**D–F**) under different ambient temperatures. **A** and **D** Glucocorticoid receptor mRNA expression level. **B** and **E** Thyroid hormone receptor alpha mRNA expression level. **C** and **F** Thyroid hormone receptor beta mRNA expression level. Data are expressed as mean ± SEM (*n*=8). ^*^P < 0.05, ^**^P < 0.01, ^***^P < 0.001

As shown in Fig. 5, compared with that of broilers reared under RT conditions, the expression of *THRβ* (*P*_temperature_ = 0.004) in the liver, and *GR* (*P*_temperature_ = 0.003) and *THRα* (*P*_temperature_ < 0.001) in the breast muscle of broilers under HT conditions were markedly upregulated*.* Moreover, the results of multiple comparisons analysis showed that the expression of *THRβ* (*P* = 0.004) in the liver, along with the expression of *GR* (*P* = 0.002), *THRα* (*P* < 0.001) and *THRβ* (*P* = 0.006) in the breast muscle, were increased in control chickens exposed to HT. However, this effect was attenuated by microbiota intervention. Compared with the control, ABX treatment prominently suppressed the expression of *GR* (*P*_ABX_ = 0.019), *THRα* (*P*_ABX_ = 0.033) and *THRβ* (*P*_ABX_ = 0.002) in the liver and *THRβ* (*P*_ABX_ = 0.010) in the breast muscle of chickens. Specifically, under RT conditions, there was no notable difference (*P* > 0.05) in the expression of *GR*, *THRα* and *THRβ* in either breast muscle or the liver between the control and ABX groups. Under HT conditions, the expression of *THRβ* (*P* = 0.032) in the liver and *GR* (*P* = 0.025), *THRα* (*P* = 0.028), and *THRβ* (*P* < 0.001) in the breast muscle was significantly decreased in the ABX-treated chickens.

**Fig. 6:**
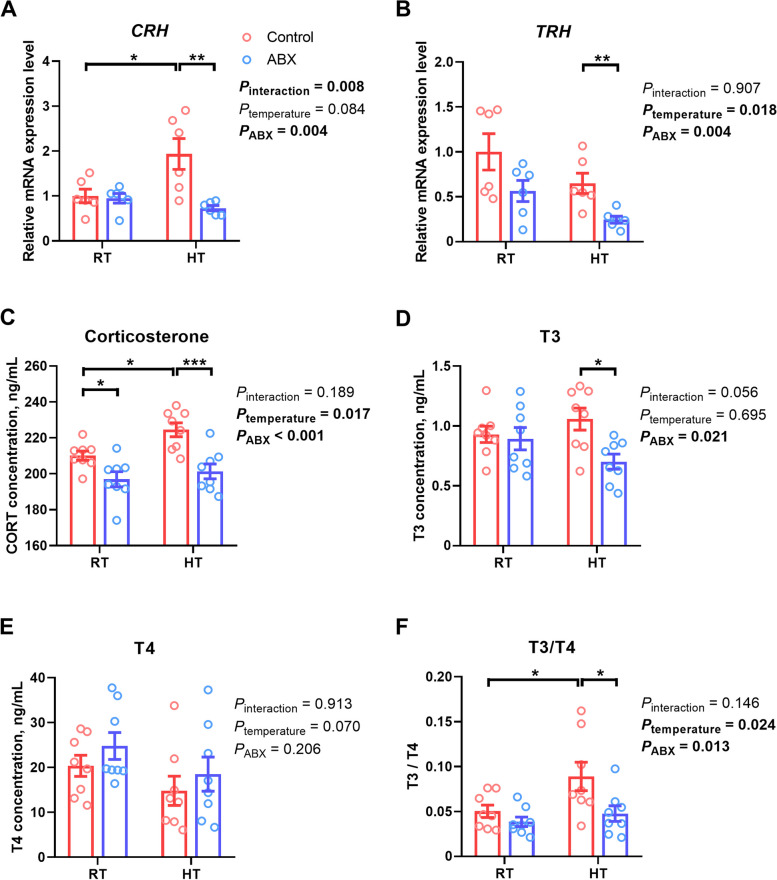
Effect of gut microbiota intervention on HPA/HPT axis activation under different ambient temperatures. **A** Corticotropin-releasing hormone mRNA expression level in the hypothalamus. **B** Thyrotropin-releasing hormone mRNA expression level in the hypothalamus. **C** Corticosterone concentration in serum. **D** T3 concentration in serum. **E** T4 concentration in serum. **F** T3/T4 ratio in serum. Data are expressed as mean ± SEM (*n *= 6 or 8). ^*^P < 0.05, ^**^P < 0.01, ^***^P < 0.001

In the hypothalamus (Fig. 6), the expression of *TRH* (*P*_temperature_ = 0.018) was significantly reduced following HT exposure compared to that under RT conditions, whereas the expression of *CRH* (*P*_temperature_ = 0.084) trended toward an increase. Particularly, exposure to HT led to an increase in the expression of *CRH* (*P* = 0.015) in control broilers, while this effect was not observed in ABX-treated chickens. In contrast, ABX treatment effectively altered the expression of both *CRH* (*P*_ABX_ = 0.004) and *TRH* (*P*_ABX_ = 0.004). Specifically, under RT conditions, no significant difference (*P* > 0.05) was observed in the expression of *CRH* and *TRH* between the control and ABX groups. However, under HT conditions, the expression of both *CRH* (*P* = 0.001) and *TRH* (*P* = 0.006) was notably suppressed by ABX treatment.

Furthermore, the concentration of hormones related to the HPA/HPT axis in the serum was measured (Fig. 6). In comparison with RT, HT exposure elevated the corticosterone concentration (*P*_temperature_ = 0.017) and the T3/T4 ratio (*P*_temperature_ = 0.024) in the serum and trended toward a decrease in the T4 concentration (*P*_temperature_ = 0.070). In detail, the corticosterone concentration (*P* = 0.049) and the T3/T4 ratio (*P* = 0.049) were increased only in control chickens, whereas ABX chickens were unaffected (*P* > 0.05) by HT. Moreover, ABX treatment significantly decreased the corticosterone (*P*_ABX_ < 0.001) and T3 (*P*_ABX_ = 0.021) concentrations, as well as the T3/T4 ratio (*P*_ABX_ = 0.013). Under RT conditions, ABX treatment resulted in a reduction in only corticosterone levels (*P* = 0.017) in the serum. Under HT conditions, the corticosterone level (*P* < 0.001), T3 content (*P* = 0.019), and T3/T4 ratio (*P* = 0.032) were all reduced in the ABX treatment group compared to those in the control group.

### Effects of microbiota intervention on microbial diversity in the cecum of broilers under different ambient temperatures

**Fig. 7:**
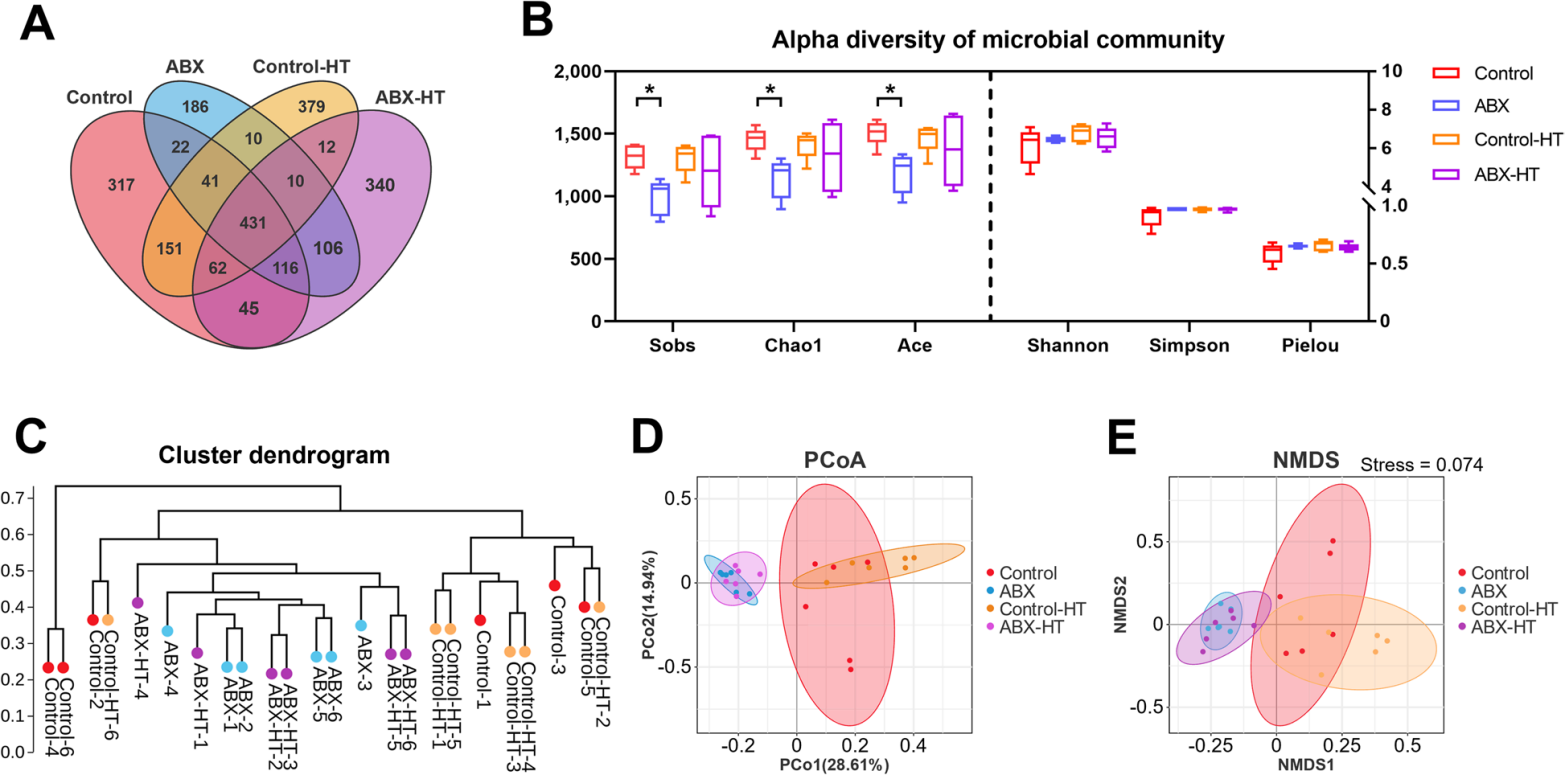
Changes of microbial diversity in the cecum of broilers. **A** Venn diagram of the operational taxonomic units (OTUs) among all groups. **B** Cecum bacterial richness (Sob1, ACE and Chao1) and diversity (Shannon, Simpson and Pielou) were evaluated by unpaired *t*-test analysis. **C** The cluster dendrogram was drawn to reveal the distance between all samples. **D** Principal coordinate analysis (PCoA) was conducted at the OTU level. **E** Non-metric multidimensional scaling (NMDS) analysis was conducted at the OTU level. Stress = 0.074, *P* = 0.008. Data are expressed as the mean ± SEM (*n =* 6). ^*^P < 0.05

The cecal microbiota was analyzed by sequencing the bacterial 16S rRNA V3+V4 region (Fig. 7). Clean tags were obtained and subjected to subsequent analysis after filtering the lower-quality reads. All the effective tags were then clustered into OTUs based on a 97% similarity level. The Venn diagram showed that there were 1,185 OTUs in the control group, of which 317 OTUs were unique; the ABX group had 922 OTUs, of which 186 were unique; the control-HT group had 1,096 OTUs, of which 379 were unique; and the ABX-HT group had 1,122 OTUs, of which 340 were unique. The cecum microbial community richness was considerably decreased in the ABX group compared to that in the control group, as indicated by the Sobs (*P* = 0.026), Chao1 (*P* = 0.030), and Ace (*P* = 0.026) indices. Microbiota diversity showed no significant differences (*P* > 0.05) between the control and ABX groups, as illustrated by the Shannon, Simpson and Pielou indices. There was no significant difference (*P* > 0.05) in the cecum microbial community richness or diversity between the control-HT and ABX-HT groups. A cluster dendrogram was used to determine the distance between all samples. Based on clustering, PCoA and NMDS results at the OTU level were plotted to evaluate the β diversity among all 4 groups. The results of both PCoA and NMDS analyses indicated a significant separation in the cecum microbial composition of the ABX and ABX-HT groups compared to that of the control or control-HT group. Additionally, a slight separation was observed between the control and control-HT groups, while no significant differences were observed between the ABX and ABX-HT groups.

**Fig. 8:**
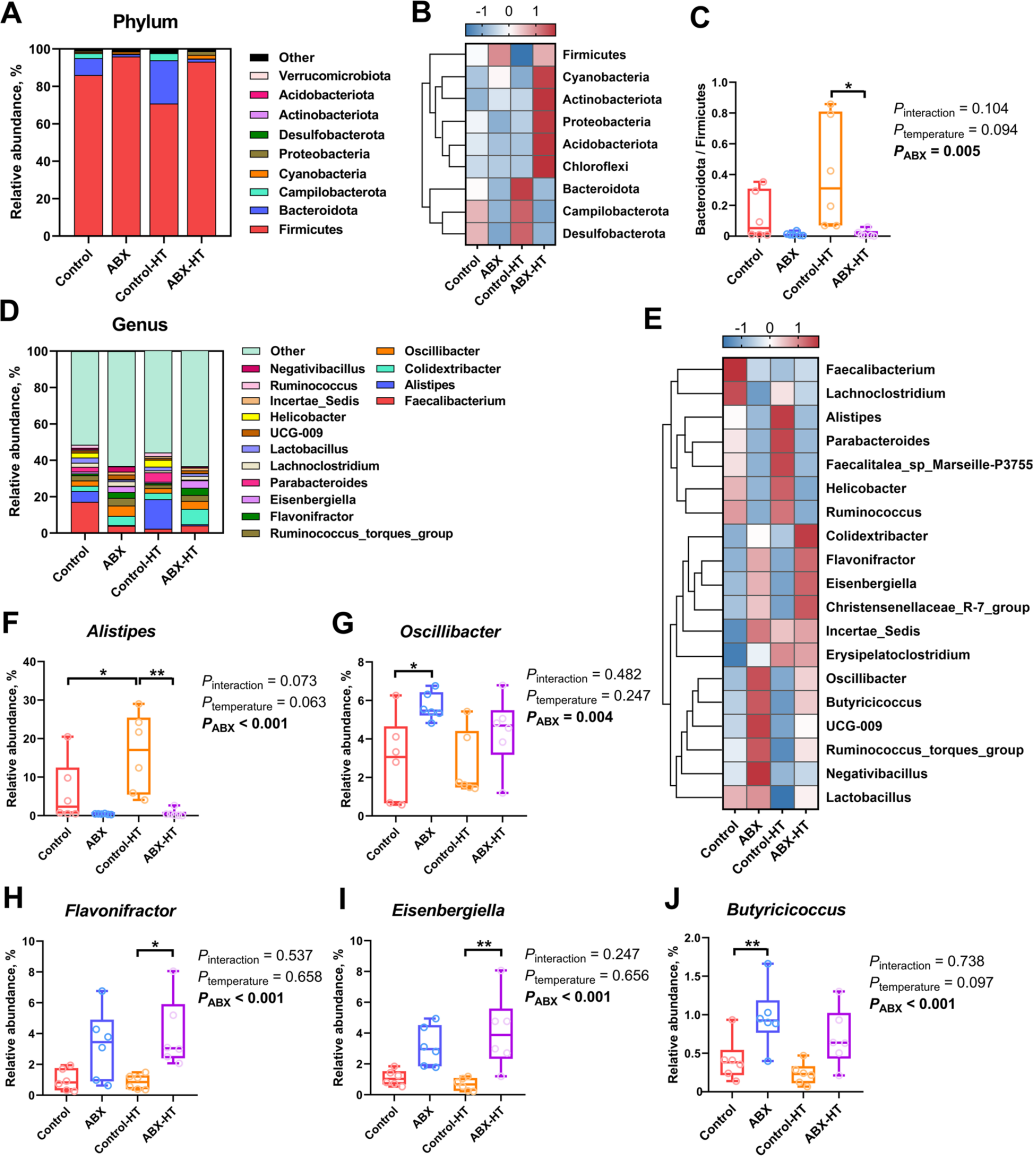
Changes of microbial community composition in the cecum of broilers. **A** Distribution of cecal microbiota at the phylum level. **B** Heatmap of species relative abundance of cecal microbiota at the phylum level. **C** The ratio of Bacteroidetes and Firmicutes was calculated. **D** Distribution of cecal microbiota at the genus level. **E** Heatmap of species relative abundance of cecal microbiota at the genus level. **F–J** The top 5 dominant genera with significant differences among all groups. Data are expressed as the mean ± SEM (*n =* 6). ^*^P < 0.05, ^**^P < 0.01, ^***^P < 0.001

The microbial communities in the cecum of broiler chickens in each group were analyzed at the phylum and genus levels (Fig. 8). At the phylum level, Firmicutes and Bacteroidota were the dominant phyla in the cecum community of AA broiler chickens at 28 days of age, accounting for approximately 80% of the total cecum bacterial community. A heatmap revealed that the relative abundance of Firmicutes was higher in the ABX-HT group, while the abundance of Bacteroidota was lower, compared to those in the control-HT group. Likewise, the ratio of Bacteroidota/Firmicutes was markedly decreased (*P* = 0.012) in the ABX-HT group. The predominant genera were *Faecalibacterium*, *Alistipes*, *Colidextribacter*, *Oscillibacter*, *Ruminococcus_torques_group*, *Eisenbergiella*, *Lachnoclostridium*, and so on. The top 5 dominant genera exhibiting differences among all groups were identified and characterized. Compared to RT conditions, exposure to HT tended to elevate the abundance of *Alistipes* (*P*_temperature_ = 0.063) while reducing that of *Butyricicoccus* (*P*_temperature_ = 0.097). Conversely, ABX treatment resulted in a notable decrease in the relative abundance of *Alistipes* (*P*_ABX_ < 0.001) while simultaneously fostering a relative enrichment of *Oscillibacter* (*P*_ABX_ = 0.004), *Flavonifractor* (*P*_ABX_ < 0.001), *Eisenbergiella* (*P*_ABX_ < 0.001), and *Butyricicoccus* (*P*_ABX_ < 0.001). At the genus level, compared to those in the control group, the relative abundance of *Alistipes* was significantly increased (*P* = 0.047) in the control-HT group, and the relative abundances of *Oscillibacter* (*P* = 0.049) and *Butyricicoccus* (*P* = 0.029) were elevated in the ABX group. In comparison with the control-HT group, the ABX-HT group exhibited a significant decrease in the relative abundance of *Alistipes* (*P* = 0.002), accompanied by an increase in the relative abundance of *Flavonifractor* (*P* = 0.018) and *Eisenbergiella* (*P* = 0.002).

**Fig. 9:**
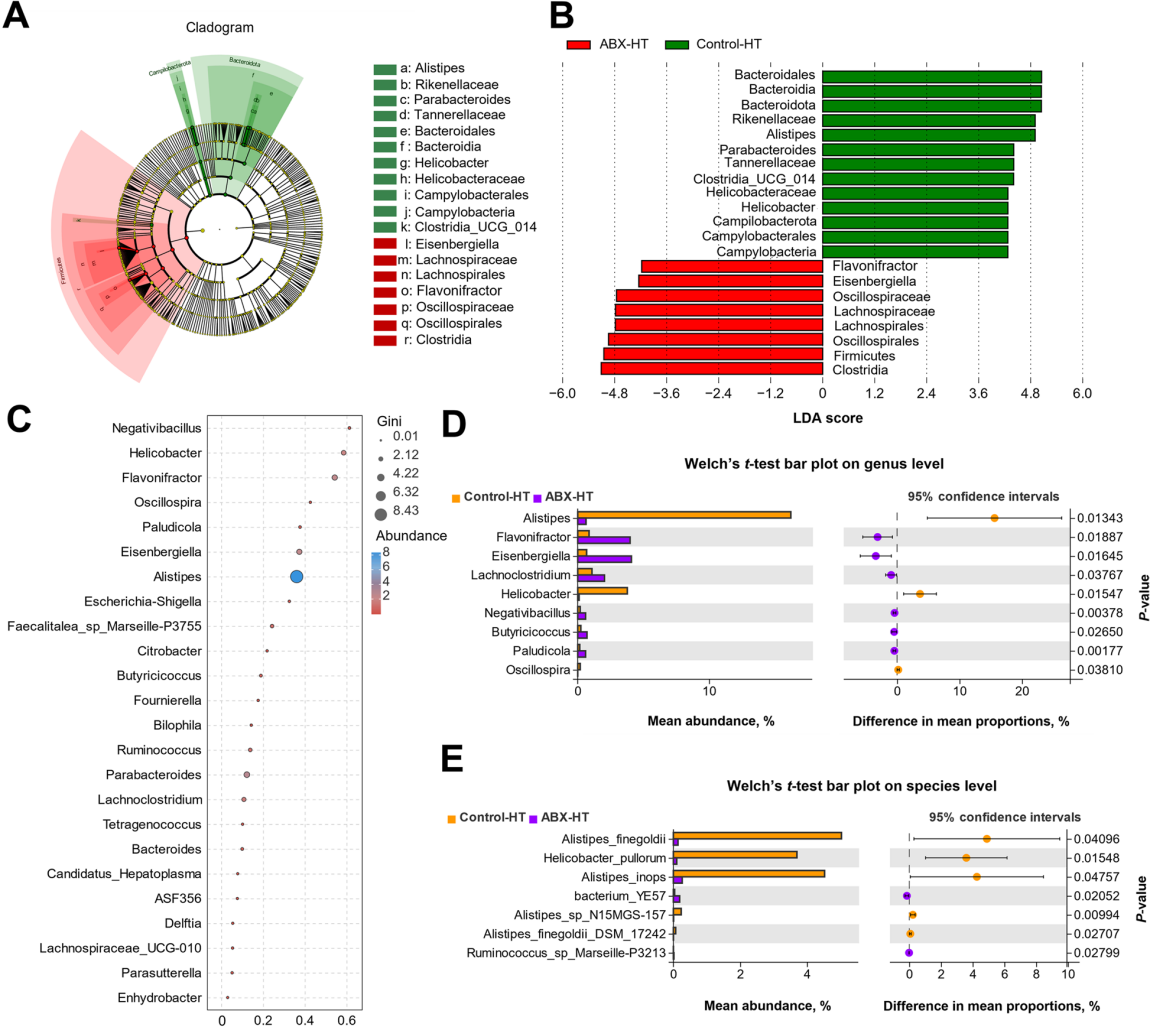
Differences in the characteristic cecal microbiota of broiler chickens between the control-HT and ABX-HT groups. **A** Taxonomic cladogram generated from LEfSe. Green indicates enriched taxa in the control-HT group. Red indicates enriched taxa in the ABX-HT group. **B** Bar chart showing the LDA score of bacterial taxa (LDA > 4). Bacterial taxa covered from phylum to genus level. **C** The bubble chart generated from Random Forest analysis revealed the Gini index and relative abundance of bacteria at the genus level. **D** and **E** The Welch's *t*-test bar chart shows the bacteria with significant differences in relative abundance between control-HT and ABX-HT groups at the genus (**D**) and species (**E**) levels. Significant differences were defined as *P* < 0.05

The specific bacterial taxa that differed in abundance between the control-HT and ABX-HT groups were identified using linear discriminant analysis effect size (LEfSe, LDA score > 4.0). Analysis with the LEfSe method revealed that the control-HT group was characterized by Bacteroidota and related genera such as *Parabacteroides* and *Alistipes*, whereas several microbes belonging to the order Firmicutes, such as *Lachnospirales* and *Oscillospirales*, were considered as the critical genera in the ABX-HT group (Fig. 9). The results of a random forest analysis indicated that *Alistipes* had the highest Gini index and was considered a potential indicator species between the control-HT and ABX-HT groups. The results of Welch's *t-*test indicated a significant reduction in the abundances of *Alistipes*, *Helicobacter*, and *Oscillospira* and an elevation in the abundances of *Flavonifractor*, *Eisenbergiella*, *Lachnoclostridium*, *Negativibacillus*, *Butyricicoccus*, and *Paludicola* in the ABX-HT group relative to those in the control-HT group. Specifically, at the species level, broiler chickens in the ABX-HT group showed a reduced abundance of *Alistipes_finegoldii*, *Alistipes_inops*, *Alistipes_sp_N15MGC_157*, and *Alistipes_finegoldii_DSM_17242* in their cecal microbiota. Spearman's correlation analysis was performed to explore the relationships of predominant cecal genera and species with rectal temperature, thermogenesis, and hormone release (Fig. S1, additional file 3). Regarding differential bacterial genera, *Alistipes* and *Helicobacter* abundances were significantly positively correlated with rectal temperature, *avUCP* expression and hormone release, whereas *Flavonifractor*, *Eisenbergiella*, *Lachnoclostridium*, *Negativibacillus,* and *Butyricicoccus* abundances were significantly negatively correlated with rectal temperature. At the species level, *Alistipes_finegoldii*, *Alistipes_inops*, *Alistipes_sp_CHKCI003*, *Alistipes_sp_N15MGC_157*, and *Helicobacter_pullorum* abundances exhibited significant positive correlations with rectal temperature and thermogenesis. On the other hand, *Ralstonia_insidiosa* and *bacterium_YE57* abundances displayed significant negative correlations with rectal temperature.

### Effect of microbiota intervention on the hypothalamic 5-HT metabolism of broilers under different ambient temperatures

**Fig. 10:**
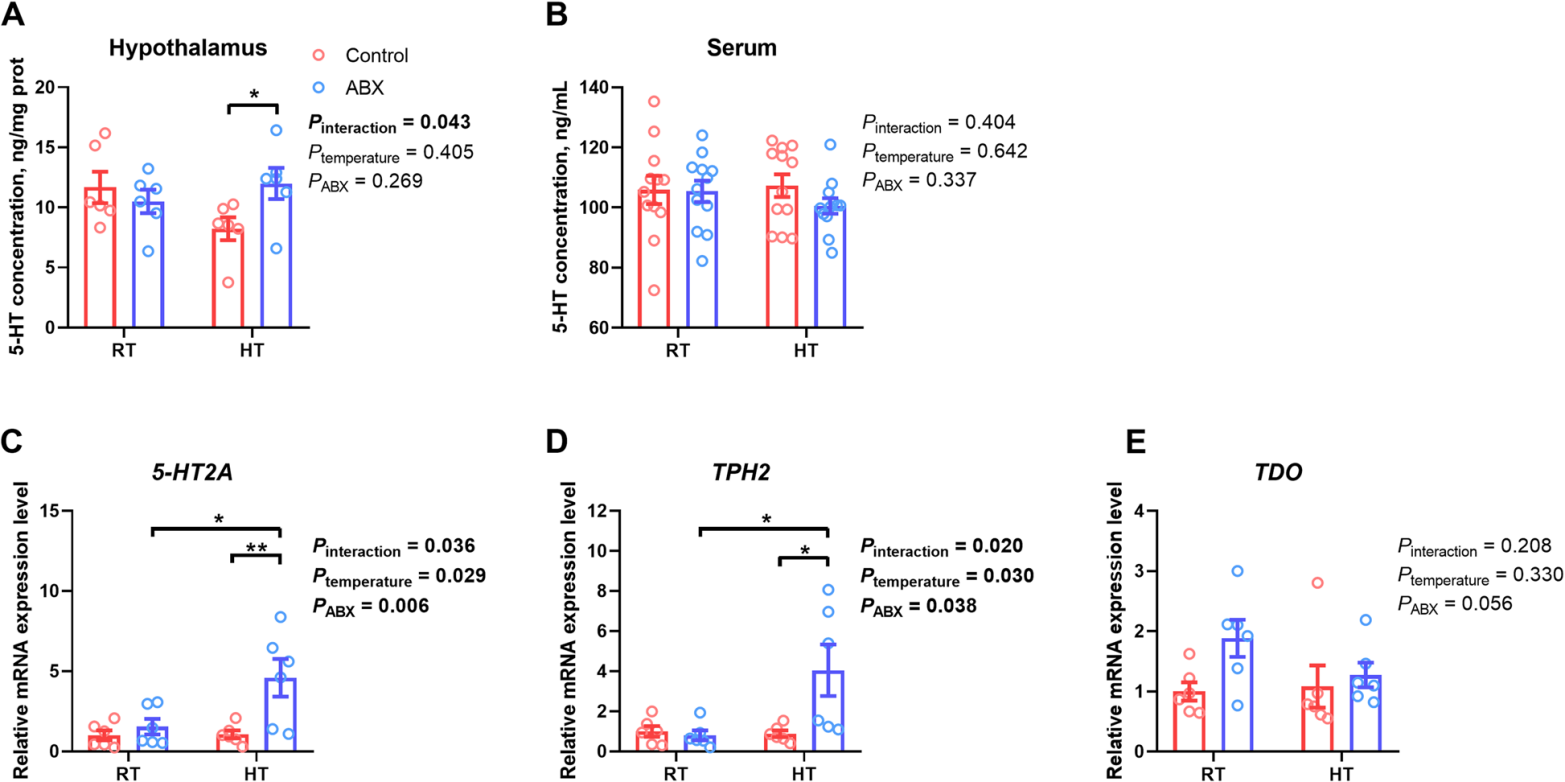
Effect of gut microbiota intervention on 5-hydroxytryptamine (5-HT) metabolism pathway in the hypothalamus under different temperature conditions. **A** 5-HT concentration in the hypothalamus (*n =* 6). **B** 5-HT concentration in serum (*n =* 12). **C**–**E** Expression of hypothalamic 5-HT metabolism-related genes (*n =* 6). **C** 5-HT receptor 2A mRNA expression level. **D** Tryptophan hydroxylase 2 mRNA expression level. **E** Tryptophan-2,3-dioxygenase mRNA expression level. Data are expressed as mean ± SEM. * *P* < 0.05, ** *P* < 0.01, *** *P* < 0.001

Compared to broiler chickens reared under RT conditions, those exposed to HT did not exhibit an impact on the 5-HT concentration in either the serum (*P*_temperature_ = 0.642) or hypothalamus (*P*_temperature_ = 0.405) but showed enhanced expression of *5-HT2A* (*P*_temperature_ = 0.029) and *TPH2* (*P*_temperature_ = 0.030) in the hypothalamus (Fig. 10). However, the enhancement in hypothalamic *5-HT2A* (*P* = 0.019) and *TPH2* (*P* = 0.013) expression induced by HT was observed solely in ABX-treated broilers, not in the control broilers. Moreover, ABX treatment significantly increased the expression of *5-HT2A* (*P*_ABX_ = 0.006) and *TPH2* (*P*_ABX_ = 0.038) compared with that in the control group. Additionally, an interactive effect between environmental temperature and ABX treatment was detected concerning hypothalamic 5-HT content (*P*_interaction_ = 0.043), as well as *5-HT2A* (*P*_interaction_ = 0.036) and *TPH2* (*P*_interaction_ = 0.020) expression. Specifically, no significant differences were observed in the levels of the aforementioned indicators under RT conditions (*P* > 0.05). However, under HT conditions, ABX treatment significantly elevated the concentration of 5-HT (*P* = 0.041) in the hypothalamus, but not in serum, while concurrently upregulating the expression of *5-HT2A* (*P* = 0.006) and *TPH2* (*P* = 0.015) compared to that with the control.

## Discussion

The relationship between the gut microbiota and the physiological states of the host has been increasingly studied and reported. In many instances, the intricate commensal microbiota inhabiting the gastrointestinal tract can notably impact the host's phenotype and its ability to adapt to variations in environmental temperatures. This study aimed to investigate the impacts and potential mechanisms of the gut microbiota intervention on the thermoregulation and thermogenesis in broiler chickens under varying environmental temperature conditions.

Antibiotics are frequently used to study the effects of the gut microbiota on the host. However, the pharmacological effects of antibiotics within the host may obscure the influence of the gut microbiota [22]. Therefore, in this study, we applied aminoglycoside antibiotics as an intervention for the gut microbiota, as they exhibit limited absorption and utilization within the body [23, 24]. We assessed the effectiveness of gut microbiota intervention induced by antibiotics in broiler chickens during the early growth phase, a critical period for gut microbiota colonization [25]. As elucidated in the results section, the ABX protocol resulted in a reduction of over 10% in the population of cecal total bacteria, confirming the successful establishment of the model. Furthermore, the administration of antibiotics increased the body weight and ADG of the chicks. This finding is consistent with previous research reporting that antibiotics are growth promoters in broiler chickens [26, 27]. We speculated that part of the reason may be that ABX greatly reduced the number of microbes in the gut of chicks, which in turn reduced competition between the microbes and the host for nutrients in the gut lumen [28, 29]. Another reason may be attributed to the reduction in the heat increment of broilers, leading to an increase in energy utilization [30, 31]. Notably, the simultaneous decrease in rectal temperature provides substantial evidence in support of this hypothesis. However, it is essential to acknowledge that during the early growth stages of broilers, the room temperature gradually decreases as the broilers grow [18, 32]. Consequently, it becomes challenging to evaluate changes in the thermoregulatory capacity of broilers during this period, given that ambient temperature significantly influences body temperature [33]. To address this, we introduced different constant temperature conditions during the later growth stages, when temperature sensitivity is more pronounced, to assess the impact of the gut microbiota on broiler thermoregulation.

However, during the later stages of growth, this difference in growth performance and rectal temperature of broilers treated with ABX disappeared under RT conditions. This phenomenon can be attributed to the gradual maturation of the thermoregulatory system, which becomes more proficient at maintaining a consistent body temperature [18, 34], thereby mitigating the impact of gut microbiota intervention. However, the thermoregulatory ability of warm-blooded animals is limited. Under certain conditions, such as high temperature or cold, the thermoregulatory system is more likely to be disrupted [35, 36]. Indeed, under HT conditions, we found that gut microbiota intervention can still induce a reduction in rectal temperature. We speculated that this may be attributable to changes in the thermogenesis of broilers. It has been reported that mice without a gut microbiota exhibit blunted UCP1 expression in brown adipose tissue and experience a faster drop in body temperature in response to cold exposure [2]. Unlike mice, broilers lack brown adipose tissue and rely on skeletal muscle and the liver for thermogenesis [15, 37]. Specifically, the abundant mitochondria in skeletal muscle and the liver use ingested nutrients for two distinct processes: producing ATP to provide energy to the body through coupled respiration (oxidative phosphorylation) or generating heat through uncoupled respiration [38–40]. On this basis, we further evaluated the expression of genes associated with mitochondrial oxidative phosphorylation and thermogenesis in the breast muscle and liver of broilers.

However, we observed no differences in the expression of genes related to oxidative phosphorylation, suggesting that gut microbiota intervention does not impact the normal process of mitochondrial energy metabolism. Notably, under HT conditions, the ABX-treated chickens exhibited a downregulation in the expression of most thermogenic genes, including *avUCP* and *PGC-1α*. avUCP is a variant of UCP1 in poultry that uncouples mitochondrial oxidative phosphorylation to generate heat from the oxidation of carbohydrates and lipids [38, 41]. PGC-1α has been reported to be associated with energy metabolism and can regulate the expression of *avUCP* genes, thereby participating in the process of thermogenesis through lipid oxidation [42]. In this context, the decreased expression of *avUCP* and lipid oxidation genes suggests that gut microbiota intervention inhibits uncoupled mitochondrial respiration, subsequently diminishing thermogenesis. This alteration constitutes a primary factor contributing to the decrease in body temperature. Conversely, the decrease in avUCP-mediated uncoupled respiration often indirectly augments the flux of coupled respiration, ultimately enhancing the efficiency of mitochondrial energy metabolism [43]. As demonstrated by increased body weight and feed conversion efficiency, gut microbiota intervention resulted in broilers allocating more energy toward growth and development rather than heat increment [29, 44]. Notably, there was no significant change in the feed intake of broilers with microbiota intervention, indicating that energy intake was not the cause for the lower energy expenditure and the dampened thermogenic capacity. In summary, our work supports the existence of a link between the gut microbiota and avUCP-dependent thermogenesis of skeletal muscle and the liver in broilers.

Ambient temperature is a critical factor influencing the body temperature and thermogenesis of broiler chickens. In fact, thermogenesis in endotherms is maintained as homeostasis under thermoneutral temperatures. However, when the environmental temperature changes, these animals require adaptive thermogenesis to respond to the new conditions. This process is more susceptible to other thermogenesis factors [45]. Unlike mice, broilers' core body temperature is closer to the upper limit of thermal comfort, making their thermoregulation capacity more fragile in HT environments [46–48]. Consistent with this, our results indicate that HT exposure reduces broiler feed intake and significantly elevates broiler body temperature. Although the reduction in feed intake is actually a protective measure employed by broilers to adapt to elevated environmental temperatures [49–51]. However, it must be mentioned that the decrease in energy supply resulting from reduced feed intake may paradoxically stimulate metabolism and thermogenesis [52]. Hence, the thermogenesis of broilers in a HT environment does not exhibit a continuous reduction but rather follows a nonlinear pattern characterized by an initial decrease followed by an increase [53]. In this study, HT exposure induced an increase in the expression of thermogenic genes *PGC-1α* and *Adrb2* in breast muscle, supporting the above point. However, these variables were significantly different between ABX-treated chickens and control chickens, suggesting that the gut microbiota is at least partially involved in the thermoregulatory process induced by ambient temperature [50]. Intriguingly, gut microbiota intervention appears to exert distinct effects on thermoregulation under varying temperature conditions. One primary explanation is the heightened vulnerability of the thermoregulatory system in broilers under HT conditions. Furthermore, body temperature itself serves as a potential influencing factor. Notably, body temperature can reciprocally impact microbial composition since the optimal growth temperature of microorganisms can differ among populations [50]. This undoubtedly amplifies the interplay between gut microbes and ambient temperature. Indeed, this study unveiled a noteworthy interaction between ambient temperature and ABX treatment concerning broiler chickens' rectal temperature and the expression of thermogenic genes in breast muscle. This interaction underscores a crosstalk between these factors. Consequently, we hypothesized that the role of the gut microbiota may hinge on the host response to environmental temperature fluctuations. As such, we conducted further investigations into the impact of gut microbiota intervention on the central thermoregulatory system.

The process of thermoregulation is intricate, with the thermogenesis of the liver and skeletal muscles relying heavily on the allocation instructions from the central nervous system [54, 55]. The hypothalamus acts as a central regulator of body temperature in broiler chickens, transmitting thermal information to peripheral thermogenic effectors such as skeletal muscles and the liver via various pathways, including the sympathetic nervous system and the HPA and HPT axes, regulating avUCP-dependent uncoupling thermogenesis in response to environmental temperature [54–56]. In the present study, HT exposure significantly increased hypothalamic *CRH* expression and the serum CORT concentration, along with the T3/T4 ratio. As the ultimate effectors of the HPA/HPT axis, these hormones interact with hormone receptors in target organs, such as GR and THR, exerting a pivotal role in the regulation of thermogenesis, energy metabolism, and various other physiological processes [17, 57]. Elevated corticosterone levels have been reported to be considered a biomarker of heat stress, as they aid in energy metabolism and expenditure under stress conditions [58, 59]. However, this heat stress response was attenuated in microbiota-intervened broilers, suggesting that gut microbiota intervention affects the balance of energy metabolism by regulating corticosterone levels through the HPA axis [60]. Furthermore, under HT conditions, gut microbiota intervention significantly suppressed the expression of *CRH* and *TRH* in the hypothalamus. This downregulation subsequently results in decreased levels of CORT and T3 (the biologically active form) downstream of the HPA/HPT axis, as well as reduced expression of *GR*, *THRα*, and *THRβ* in the breast muscle and *THRβ* in the liver. Thyroid hormones (T3 and T4) contribute to obligatory and facultative thermogenesis [57]. In terms of resting energy expenditure, thyroid hormone stimulates the transcription of *UCP* genes via the thyroid hormone receptor, resulting in increased proton gradient leaks in mitochondria and, thus, heat generation [17]. It has been reported that antibiotic treatment reduced serum T4 concentrations in mice, leading to a reduction in thermogenesis and an accelerated decline in body temperature [61]. Another study noted that piglets exposed to antibiotics exhibited reduced serum T3 concentrations without significant changes in T4 concentrations, ultimately resulting in reduced cold tolerance [62]. In line with these findings, although the T4 concentration remained unaffected, we hypothesized that microbiota intervention may have inhibited the conversion process of T4 to T3, leading to a decrease in the T3 concentration and the T3/T4 ratio and effectively reducing thyroid hormone receptor expression in skeletal muscle and the liver, further interfering with avUCP-dependent thermogenesis. It should be noted that gut microbiota themselves are an important source of peripheral neurotransmitters and hormones, and alterations in the gut microbiota can have a direct or indirect impact on hypothalamic neurons through the bidirectional connection of the gut-brain axis [60, 63]. Given the central role of the hypothalamus in thermogenesis and thermoregulation in birds, we speculated that gut microbes may regulate thermogenesis and body temperature in broiler chickens via the gut-brain axis.

To identify vital bacterial taxa regulating host thermoregulation, we comprehensively analyzed the control-HT and ABX-HT groups, which exhibited differences in body temperature and thermogenesis. The microbial communities were significantly different between the two groups, with *Alistipes* and *Parabacteroides* enriched in the control-HT group, while *Flavonifractor*, *Eisenbergiella*, and *Lachnospira* enriched in the ABX-HT group. The differences in flora may endow the two groups of broilers with different metabolic characteristics. For example, *Alistipes*, a genus within the phylum Bacteroidetes, has tryptophan metabolic activity [64]. *Parabacteroides* have physiological properties of carbohydrate metabolism and short-chain fatty acids (SCFAs) secretion [65]. *Eisenbergiella* and *Lachnospira* are members of Lachnospiraceae, which hydrolyze starch and other sugars to produce butyrate and other short-chain fatty acids [66]. *Flavonifractor* belongs to Ruminococcaceae in Firmicutes and contributes to butyrate production [67]. Surprisingly, the ABX-HT group exhibited a higher relative abundance of bacterial groups associated with SCFAs metabolism. Conversely, the abundance of *Alistipes* and other bacterial groups, which compete with the host for amino acid nutrition, was reduced. This finding means that the broilers in the ABX group had more available energy substances for growth, which may be the reason for the better growth performance than the control group broilers. Importantly, the interaction between host thermoregulation and the microbiota may be contingent on the production of various bioactive small-molecule metabolites. Lachnospiraceae has previously been observed to be enriched in the intestines of cold-exposed mice, suggesting its potential involvement in cold exposure-induced thermogenesis in mammals [2, 6]. In mice with antibiotic-induced gut microbiota depletion, the reduction in the abundance of Lachnospiraceae and its metabolite butyrate led to hypothermia, and butyric acid supplementation alleviated the sustained decrease in body temperature in mice under cold exposure [3, 4]. Additionally, cold stimulation induces an increase in the relative levels of Ruminococcaceae, and transplantation of Ruminococcaceae has been shown to promote bile acid metabolism and enhance the body's thermogenic capacity [5, 68]. In contrast, mice enriched with Bacteroidetes exhibited hypothermia and impaired thermogenesis [5]. These reports contrast with our initial hypothesis. Given the species differences, the role of these specific bacteria in broiler thermoregulation and energy metabolism deserves further investigation.

Nonetheless, considering the relative abundance and difference in the microbial communities, *Alistipes* appears to be a potential indicator species between the two groups. Importantly, although no differences in diversity were observed between RT and HT, at the genus level, only *Alistipes* exhibited an increasing trend in the HT group. The abundance of *Alistipes* appears to be influenced by variations in feed intake. *Alistipes* have been found to be significantly enriched in the intestines of mice subjected to intermittent fasting [69]. However, contrasting findings indicate that rabbits subjected to nighttime feeding restrictions exhibit reduced *Alistipes* levels [70]. Despite the inconsistent findings across various studies, considering the reduced feed intake observed in broilers under HT conditions, we hypothesized that the observed rise in *Alistipes* abundance could be linked to this factor. However, it is evident that the reduction in *Alistipes* abundance resulting from gut microbial intervention is unrelated to feed intake, as no differences in feed intake were observed between the ABX and control groups, and is more likely due to the inhibitory effect of antibiotics. Notably, alterations in *Alistipes* levels seem to play a pivotal role in driving changes in body temperature. It has been reported that in feed-restricted rabbits, body temperature oscillations are synchronized with *Alistipes* abundance and *Alistipes*-driven circadian rhythms of serotonin [70]. Coincidently, our findings indicated a significant positive correlation between almost all *Alistipes* strains and body temperature as well as thermogenesis, suggesting that alterations in *Alistipes* may be a key factor in changes in thermoregulatory capacity caused by gut microbial intervention. However, it should be noted that limited research reports currently exist on this specific aspect, limiting the availability of additional valuable references. Indeed, *Alistipes* have been implicated in influencing the development and function of neurons in the brain, thus regulating host physiology and behavior [71]. It is hypothesized that an increase in *Alistipes* abundance can disrupt the gut-brain axis due to its ability to metabolize tryptophan into indole, which can decrease the availability of 5-HT, another tryptophan metabolite [64, 71].

In this study, gut microbiota intervention did not alter serum levels of 5-HT but significantly increased the content of 5-HT and the expression of its receptor *5-HT2A* in the hypothalamus. Due to the presence of the blood‒brain barrier, peripheral 5-HT cannot enter the central nervous system, indicating that the central and peripheral 5-HT systems are completely independent. Interestingly, tryptophan, the precursor of 5-HT, can cross the blood‒brain barrier and then be metabolized to 5-HT by the catalytic transformation of central TPH2 or metabolized to kynurenine through the TDO pathway [72]. In the present study, gut microbiota intervention promoted the expression of *TPH2* without significantly affecting *TDO* expression, suggesting that the increase in central 5-HT levels is mainly attributable to the upregulation of TPH2 [73]. Thus, although 5-HT cannot cross the blood‒brain barrier directly, tryptophan and its metabolizing enzymes can increase hypothalamic 5-HT content, which might link *Alistipes* with brain function [64, 71]. In addition, the functions of central and peripheral 5-HT differ, with hypothalamic 5-HT serving as an important neurotransmitter involved in regulating physiological processes such as sleep, emotion, and body temperature [74–76]. It has been reported that the tryptophan–5-HT metabolic pathway in the hypothalamus is involved in the regulation of brain neurotransmitters, and central 5-HT administration can activate 5-HTergic neurons to cause hypothermia in mice and chicks [77, 78]. Exogenous tryptophan supplementation can also increase hypothalamic 5-HT levels, lowering the rectal temperature in broiler chickens and indicating a negative correlation between central 5-HT content and heat production [20]. Overall, these findings suggest that gut microbiota intervention-induced changes in the abundance of *Alistipes* and the content of hypothalamic 5-HT may be potential causes of thermogenesis regulation in broiler chickens.

As the ambient temperatures rise, broilers experience an imbalance between their internal heat production and heat dissipation mechanisms, leading to the inability to maintain a stable body temperature and subsequently causing heat stress, which can result in reduced production performance. Therefore, one potential solution to mitigate heat stress in broiler chickens is to modulate their thermoregulatory processes through the manipulation of the gut microbiota [50]. Indeed, within the context of antibiotic restrictions, numerous studies have highlighted the efficacy of incorporating feed additives that specifically target intestinal microorganisms and the tryptophan–5-HT pathway as a dependable strategy for enhancing livestock performance and poultry production. For instance, research has shown that adding dimethylglycine to the diet can modulate the gut microbiota of heat-stressed broilers and activate the 5-HT metabolic pathway within the gut-brain axis, thereby mitigating the decline in production performance induced by heat stress [73]. Laurate supplementation has been shown to reduce the abundance of* Alistipes* in the cecum, subsequently impacting serum metabolite concentrations and potentially conferring growth advantages to broilers [79]. Additionally, research has suggested that supplementation with L-tryptophan can alleviate stress-induced intestinal barrier dysfunction by regulating 5-HT metabolism in broilers [80]. Dietary supplementation with 5.0% tryptophan has been found to lower the rectal temperature and reduce the serum levels of inflammatory cytokines in heat-stressed broilers [20]. However, despite the development of numerous feed additives aimed at improving broiler performance through the manipulation of the gut microbiota, the precise roles of intestinal microorganisms themselves and the underlying mechanisms within the gut-brain axis remain incompletely elucidated. Further investigations are necessary to elucidate the role of *Alistipes* and 5-HT in hypothalamic thermoregulation, thereby advancing our understanding of the gut-brain axis mechanisms.

## Conclusion

Taken together, these findings highlight the important role of the gut microbiota in the thermoregulation of broilers. Antibiotic-induced intervention of the gut microbiota lowers body temperature in broilers under HT conditions by inhibiting HPA/HPT axis-related hormone release in the hypothalamus and subsequent avUCP-mediated thermogenesis in the liver and breast muscle. The underlying gut-brain axis connection is likely a significant driver of thermoregulation, and the hypothalamic 5-HT pathway represents the primary mechanism by which the gut microbiota regulates thermogenesis.

### Supplementary Information

**Additional file 1: Table S1.** The composition and nutrient levels of the experimental diets (1–28 d).

**Additional file 2: Table S2.** PCR primer sequences.

**Additional file 3: Fig. S1.** Correlations between the cecal microbiota and rectal temperature and thermogenesis. **A** At the genus level (The top 20 dominant genera). **B** At the species level (The top 20 dominant species). Red represents a positive correlation, and blue represents a negative correlation. ^*^P < 0.05, ^**^P < 0.01, ^***^P < 0.001.

## Data Availability

The datasets supporting the conclusions of this article are available in the NCBI repository, accession number PRJNA975430.

## Abbreviations

5-HT: 5-hydroxytryptamine
5-HT2A: 5-hydroxytryptamine receptor 2A
avUCP: Avian uncoupling protein
Acsl1: Long-chain acyl coenzyme A synthetase 1
ADFI: Average daily feed intake
ADG: Average daily gain
Adrb2: Adrenergic receptor beta-2
Adrb3: Adrenergic receptor beta-3
ATP5B: Mitochondrial ATP synthase 5 beta-subunit
Cidea: Cell deathinducing DFFA-like effector a
CORT: Corticosterone
COX IV: Cytochrome c oxidase subunit IV
CRH: Corticotropin-releasing hormone
Cyt c: Cytochrome c
FCR: Feed conversion ratio
GR: Glucocorticoid receptor
HPA: Hypothalamic-pituitary-adrenal
HPT: Hypothalamic-pituitary-thyroid
HT: High-temperature
IDH3α: Isocitrate dehydrogenase 3 (NAD+) α
NMDS: Non-metric multidimensional scaling
OTUs: Operational taxonomic units
PCoA: Principal coordinates analysis
PGC-1α: Peroxisome proliferator-activated receptor γ coactivator 1α
PPARα: Peroxisome proliferator-activated receptor α
PPARγ: Peroxisome proliferator-activated receptor γ
RH: Relative humidity
RT: Room-temperature
SCFAs: short-chain fatty acids
T3: Triiodothyronine
T4: Thyroid hormone
TDO: Tryptophan 2,3-dioxygenase
THRα: Thyroid hormone receptor α
THRβ: Thyroid hormone receptor β
TPH2: Tryptophan hydroxylase 2
TRH: Thyrotropin-releasing hormone
UCP1: Uncoupling protein 1
