# Supplementary material for: Gut microbiota intervention attenuates thermogenesis in broilers exposed to high temperature through modulation of the hypothalamic 5-HT pathway
Source: J Anim Sci Biotechnol. 2023 Dec 21;14:159. doi: 10.1186/s40104-023-00950-0 (PMC10734199; doi:10.1186/s40104-023-00950-0)
Supplement: Supplementary file 1 — Additional file 1: Table S1. The composition and nutrient levels of the experimental diets (1–28 d). [file 40104_2023_950_MOESM1_ESM.docx]

**Table S1** The composition and nutrient levels of the experimental diets (1–28 d)

| **Ingredients, %** | **1–21 d** | **21–28 d** |
| --- | --- | --- |
| Corn (8.5%) | 60.47 | 64.07 |
| Soybean meal (46%) | 33.74 | 28.84 |
| Soybean oil | 1.50 | 3.00 |
| Powder | 1.04 | 1.17 |
| Calcium hydrogen phosphate | 2.00 | 1.85 |
| Salt | 0.32 | 0.29 |
| Lysine (99%) | 0.17 | 0.15 |
| Methionine (98%) | 0.25 | 0.18 |
| Choline chloride (50%) | 0.26 | 0.20 |
| Vitamin premix feed^*^ | 0.05 | 0.05 |
| Trace element feed^†^ | 0.20 | 0.20 |
| Calculated nutrient composition | |  |
| Metabolic energy, kcal/kg | 2900 | 3030 |
| Crude protein, % | 21.0 | 19.0 |
| Lysine, % | 1.10 | 0.97 |
| Calcium, % | 0.90 | 0.90 |
| Non phytate phosphorus, % | 0.45 | 0.42 |
| Threonine, % | 0.82 | 0.74 |
| Tryptophan, % | 0.26 | 0.23 |
| Leucine, % | 1.52 | 1.42 |
| Isoleucine, % | 0.83 | 0.74 |
| Valine, % | 0.94 | 0.85 |

^*^Vitamin premix provides the following per kg of diet: VA, 8,000 IU; VD_3_, 3,000 IU; VE, 20 IU; VK, 2mg; VB_1_, 4mg; riboflavin, 8 mg; D-pantothenic acid, 11 mg; VB_5_, 40 mg; VB_6_, 4 mg; VB_12_, 0.02 mg; biotin, 0.15 mg; folic acid, 1.0 mg; choline, 700 mg

^†^Mineral premix provides the following per kg of diet: Fe (as ferrous sulfate), 80 mg; Zn (as zinc sulfate), 75 mg; Mn (as manganese sulfate), 80 mg; Cu (as copper sulfate) 10 mg, I (as potassium iodide), 0.40 mg; and Se (as sodium selenite), 0.30 mg
