# Supplementary material for: Gut microbiota intervention attenuates thermogenesis in broilers exposed to high temperature through modulation of the hypothalamic 5-HT pathway
Source: J Anim Sci Biotechnol. 2023 Dec 21;14:159. doi: 10.1186/s40104-023-00950-0 (PMC10734199; doi:10.1186/s40104-023-00950-0)
Supplement: Supplementary file 2 — Additional file 2: Table S2. PCR primer sequences. [file 40104_2023_950_MOESM2_ESM.docx]

**Table S2** PCR primer sequences

| **Gene** | **Gene Bank Number**  ***Gallus gallus*** | **Primer sequences (5′→3′)** | **Product size, bp** | **Melting temperature, ℃** |
| --- | --- | --- | --- | --- |
| *avUCP* | NM_001397652.1 | Forward: GCAGCGGCAGATGAGCTT | 62 | 91 |
|  |  | Reverse: AGAGCTGCTTCACAGAGTCGTAGA |  |  |
| *PGC-1α* | XM_046916275.1 | Forward: GACTCAGGTGTCAATGGAAGTG | 272 | 85 |
|  |  | Reverse: ATCAGAACAAGCCCTGTGGT |  |  |
| *PPARα* | XM_046906400.1 | Forward: AGACACCCTTTCACCAGCATCC | 167 | 81 |
|  |  | Reverse: AACCCTTACAACCTTCACAAGCA |  |  |
| *PPARγ* | XM_046925952.1 | Forward: CCAGCGACATCGACCAGTT | 145 | 83 |
|  |  | Reverse: GGTGATTTGTCTGTCGTCTTTCC |  |  |
| *Adrb2* | XM_040683024.2 | Forward: TGCCTCCTCCATCATCTCCTTCTAC | 149 | 83 |
|  |  | Reverse: CATTCTGGTCCTGCTCCTTGTTCTG |  |  |
| *Adrb3* | XM_040689345.2 | Forward: GTGTGACGGCGAGCATTGAGAC | 150 | 88 |
|  |  | Reverse: AGGAGATGAAGGCGGAGATGGC |  |  |
| *Acsl1* | XM_046916038.1 | Forward: CGCACCCTTCCGACAAATACCC | 144 | 83 |
|  |  | Reverse: AGCATCCTCTTCACCCTCTACCTTC |  |  |
| *Cidea* | NM_001195123.2 | Forward: TTCCGTGTGTCAAATGCCTCCAG | 150 | 83 |
|  |  | Reverse: TGTGTCCACAACTGTGCCATCTTC |  |  |
| *IDH3α* | XM_046924760.1 | Forward: TGCTGGATTGATTGGAGGTCTTGG | 94 | 80 |
|  |  | Reverse: AGGTGCTGTTCCATGAACCGATTC |  |  |
| *COX IV* | XM_046925583.1 | Forward: AAGCAGACGATTTCAGCCATCCAG | 132 | 83 |
|  |  | Reverse: AAGCGGTCCAAGATGCCTTTTCC |  |  |
| *Cyt c* | NM_001398298.1 | Forward: AATGTTCCCAGTGCCATACGGTTG | 99 | 83 |
|  |  | Reverse: AGCCCTCAGCTTGTCCTGTTTTG |  |  |
| *ATP5B* | NM_001031391.3 | Forward: AGAGATGAGCGTCGAGCAGGAG | 186 | 86 |
|  |  | Reverse: ACACCAGCGAACACCGAATAACC |  |  |
| *GR* | XM_046927041.1 | Forward: AACCTGCTCTGGCTGACTTCTC | 121 | 82 |
|  |  | Reverse: CCCATCACTTTCGCATCTGTTT |  |  |
| *THRα* | XM_046933303.1 | Forward: GCCGTGCTGCTCATGTCCTC | 123 | 79 |
|  |  | Reverse: GGGAATGTTGTGTTTGCGGTAGTTG |  |  |
| *THRβ* | NM_001252221.2 | Forward: CCCAGCTGCTGGTAGCAATT | 92 | 84 |
|  |  | Reverse: CCTGAGCATCAACACTGCTGTA |  |  |
| *CRH* | NM_001123031.1 | Forward: CTCCCTGGACCTGACTTT | 117 | 86 |
|  |  | Reverse: CCTCACTTCCCGATGATT |  |  |
| *TRH* | XM_046926189.1 | Forward: AGGCTACCTTGTCAACTACCTGGAG | 140 | 83 |
|  |  | Reverse: TTCTGCCTGGATGCTGTCTTTTGG |  |  |
| *5-HT2A* | XM_046908587.1 | Forward: AAAGTCCGCCGTGCTACTC | 250 | 83 |
|  |  | Reverse: AGAGGCCACTTGTATCCGTA |  |  |
| *TPH2* | NM_001001301.2 | Forward: GGACCTCCGCAGTGATCTAA | 203 | 81 |
|  |  | Reverse: TACACAATGACACAAGCCGC |  |  |
| *TDO* | XM_040670682.2 | Forward: GCTTACCCAACCTACTGC | 133 | 79 |
|  |  | Reverse: CTCGGATACCTTCTTGCTAC |  |  |
| *β-actin* | NM_205518.2 | Forward: CTGGCACCTAGCACAATGAA | 123 | 80 |
|  |  | Reverse: CTGCTTGCTGATCCACATCT |  |  |
