# Supplementary figures and images for: Gut microbiota intervention attenuates thermogenesis in broilers exposed to high temperature through modulation of the hypothalamic 5-HT pathway
Source: J Anim Sci Biotechnol. 2023 Dec 21;14:159. doi: 10.1186/s40104-023-00950-0 (PMC10734199; doi:10.1186/s40104-023-00950-0)

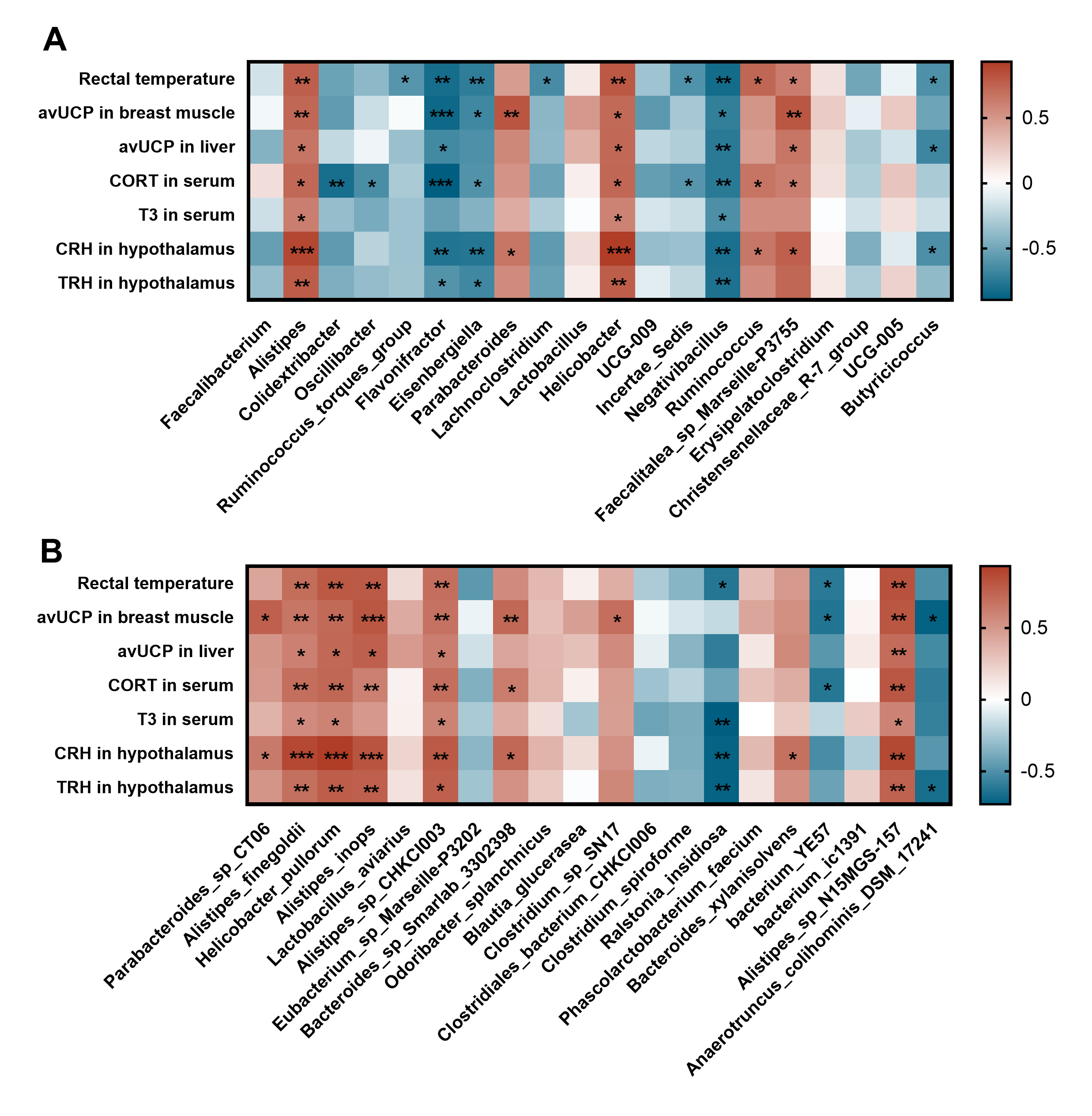

Supplement: Supplementary file 3 — Additional file 3: Fig. S1. Correlations between the cecal microbiota and rectal temperature and thermogenesis. A At the genus level (The top 20 dominant genera). B At the species level (The top 20 dominant species). Red represents a positive correlation, and blue represents a negative correlation. *P < 0.05, **P < 0.01, ***P < 0.001. [file 40104_2023_950_MOESM3_ESM.tif]
